# Bund removal to re-establish tidal flow, remove aquatic weeds and restore coastal wetland services—North Queensland, Australia

**DOI:** 10.1371/journal.pone.0217531

**Published:** 2020-01-24

**Authors:** Brett N. Abbott, Jim Wallace, David M. Nicholas, Fazlul Karim, Nathan J. Waltham

**Affiliations:** 1 CSIRO Land and Water, Australian Tropical Science and Innovation Precinct, Townsville, Australia; 2 Centre for Tropical Water & Aquatic Ecosystem Research (TropWATER), James Cook University, Townsville, Australia; 3 Independent Consultant, Townsville, Australia; 4 CSIRO Land and Water, Black Mountain Laboratories, Canberra, Australia; University of Waikato, NEW ZEALAND

## Abstract

The shallow tidal and freshwater coastal wetlands adjacent to the Great Barrier Reef lagoon provide a vital nursery and feeding complex that supports the life cycles of marine and freshwater fish, important native vegetation and vital bird habitat. Urban and agricultural development threaten these wetlands, with many of the coastal wetlands becoming lost or changed due to the construction of artificial barriers (e.g. bunds, roads, culverts and floodgates). Infestation by weeds has become a major issue within many of the wetlands modified (bunded) for ponded pasture growth last century. A range of expensive chemical and mechanical control methods have been used in an attempt to restore some of these coastal wetlands, with limited success. This study describes an alternative approach to those methods, investigating the impact of tidal reinstatement after bund removal on weed infestation, associated changes in water quality, and fish biodiversity, in the Boolgooroo lagoon region of the Mungalla wetlands, East of Ingham in North Queensland. High resolution remote sensing, electrofishing and in-water logging was used to track changes over time– 1 year before and 4 years after removal of an earth bund. With tides only penetrating the wetland a few times yearly, gross changes towards a more natural system occurred within a relatively short timeframe, leading to a major reduction in infestation of olive hymenachne, water hyacinth and salvina, reappearance of native vegetation, improvements in water quality, and a tripling of fish diversity. Weed abundance and water quality does appear to oscillate however, dependent on summer rainfall, as changes in hydraulic pressure stops or allows tidal ingress (fresh/saline cycling). With an estimated 30% of coastal wetlands bunded in the Great Barrier Reef region, a passive remediation method such as reintroduction of tidal flow by removal of an earth bund or levee could provide a more cost effective and sustainable means of controlling freshwater weeds and improving coastal water quality into the future.

## Introduction

Coastal floodplains around the world have been modified for human gain, most notably being hydrologically altered either totally or partially reducing connectivity between floodplains and coastal areas [1, 2]. Floodplain, coastal tidal and freshwater wetlands are essential habitat because they provide important biodiversity, hydrological, cultural and economic goods and services [3–5]. However, these wetlands are under great pressure due to urban and industrial development [6, 7] or agricultural and grazing land expansion, with many coastal wetlands becoming lost due to the construction of artificial barriers (e.g. bunds, roads, culverts and floodgates). These have stopped or reduced tidal flushing, which has negatively impacted aesthetic and ecological values [8]. The widespread degradation of coastal wetlands has led to major shifts in species assemblages and declines in aquatic species productivity. In response, there has been increased effort to rehabilitate coastal wetlands by removing these artificial barriers [9, 10], and provide protection and restoration of coastal wetlands [11]. The ultimate goal is to improve ecosystem services including their connectivity and functionality as productive fish habitat [12, 13] and to deliver opportunities for carbon sequestration and storage [14, 15].

The coastal wetlands of north Queensland contain unique and valuable biodiversity at the interface between two World Heritage areas; the Great Barrier Reef and Australia’s tropical rainforests [16, 17]. These wetlands are important ecological assets with significant cultural and economic values [18]. In their natural state they provide habitat for native plants, animals and migratory birds as well as potential water quality improvement and hydrology regulatory functions [19–21]. The aboriginal peoples of Australia associate great cultural value to wetlands [22, 23], which also have commercial and recreational value [24, 25]. However, many of the wetlands along the north Queensland coast, and the services they provide, have been lost; for example, it has been estimated that between 60 and 90% of freshwater and saline wetlands have vanished in favour of rural and urban development [6, 26, 27]. Of the wetlands that remain, many are degraded via a combination of earth bunding, to exclude seawater and reclaim land for pasture [28–30], upstream agricultural use (grazing and sugar cane production) which leaches ecologically damaging nutrients and sediments [31, 32], and extensive aquatic invasive weed chokes [33, 34] which contributes to hypoxic conditions and fish kills [35, 36].

The Mungalla wetlands east of Ingham, on the north Queensland coast, are characteristic of the many degraded intertidal wetlands adjacent to the Great Barrier Reef lagoon [33]. The degradation began after an earth bund was constructed in the mid-1940s, initially to provide access across the wetlands [37], which excluded seawater and created a ponded pasture for grazing [22]. A short time later para grass (*Urochloa mutica*) was introduced into the freshwater ponded area, which formed above the bund. Para grass was introduced into Queensland in 1884 for improving river bank stabilization [38] and has been used as ponded pasture in bunded areas of coastal marine plains since artificial ponding for grazing started in Queensland in the 1930’s [39]. More recently two other ponded pasture species have been introduced—olive hymenachne (*Hymenachne amplexicaulis)* and Aleman grass (*Echinochloa polystachya)*. These two species were introduced into Queensland in 1988 [40] despite apparent warning of possible weedy invasion by the Queensland Environmental Protection Agency [41, 42]. Subsequently the two grasses have been recognized regionally as weeds, with olive hymenachne being declared a weed of national significance (WONS) 10 years after release [43]. Aside from the pasture introductions, two other nationally declared weeds are present in the wetlands, salvinia (*Salvinia molesta)*, and water hyacinth (*Eichhornia crassipes*), both of which are spread by spore/seed or asexually through fragmentation. Weed growth and expansion into the wetland was exacerbated by Palm Creek carrying nutrients downstream from a large area of sugar cane and a sugar mill [33]. In response, the Mungalla Wetland Management Strategy [44] prescribed weed control using aerial and ground based application of chemical herbicides in the first instance with the possibility of exploring bund removal. The initial efforts were expensive and ecologically undesirable as freshwater weed removal was partial and short-lived. With the limited success of the chemical weed control, it was decided to investigate bund removal as a natural form of weed control using tidal ingress from the seaward side of the wetland. There was however, some uncertainty around the frequency, duration and extent of seawater penetration into the wetland once the bund was removed. Hydro-dynamic modelling simulations by Karim *et al*., [45] predicted that large (king) tides occurring in December/January and June/July each year should penetrate upstream of the existing bund. However, these simulations took no account of the impact of different depths of standing water within the wetland, which could form a hydrological barrier to, and potentially inhibit, on-going ingress of seawater. Despite these uncertainties it was decided to remove sections of the earth bund wall.

**Fig 1:**
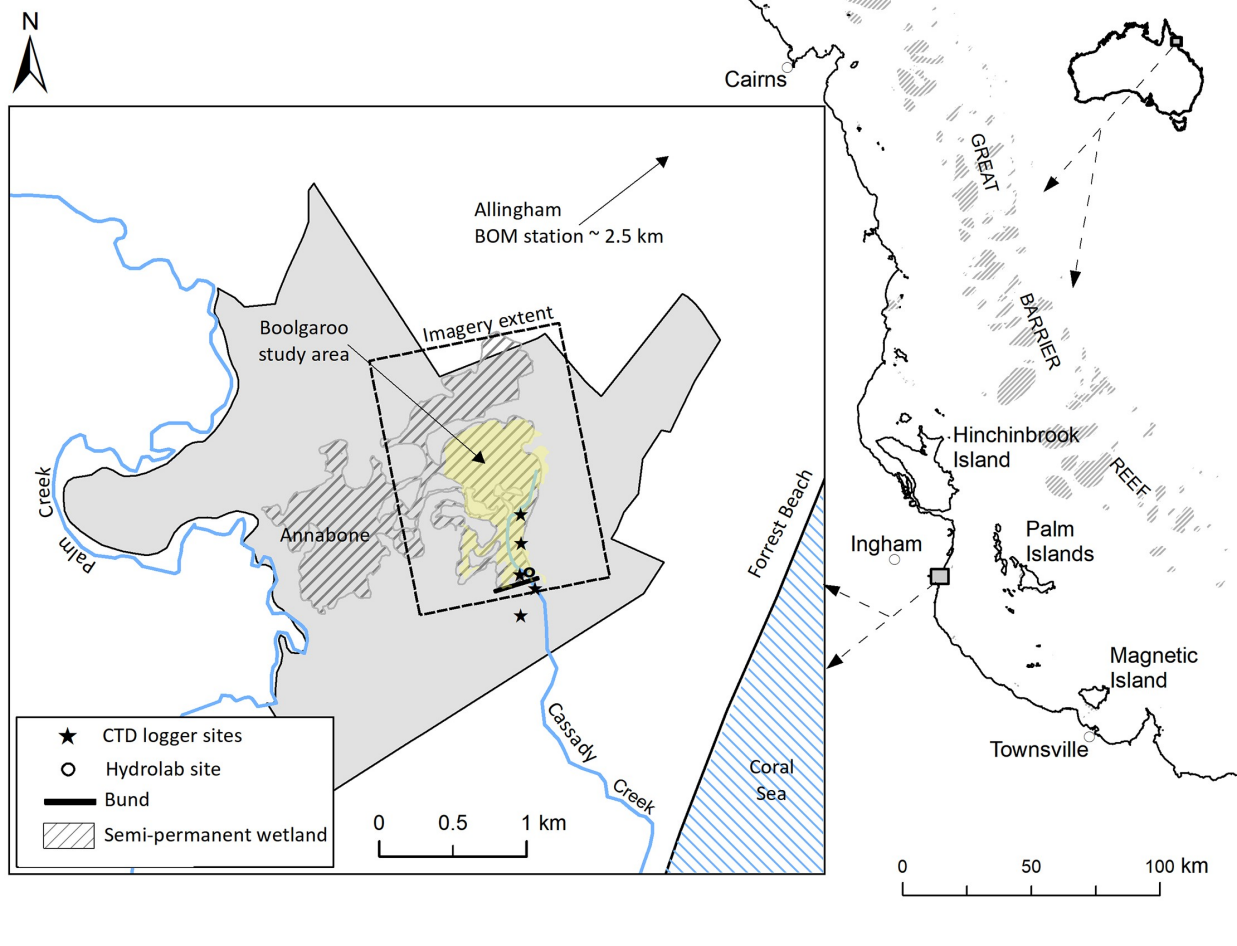
Location of wetlands within Mungalla station in the lower Herbert River catchment, Queensland, Northern Australia. The Mungalla wetlands are hatched in dark grey, the Boolgooroo region of the wetland complex is shown in yellow. Also shown are the locations of the logger sites above and below the earth bund which was removed on 6^th^ October 2013.

This paper gives an overview of the changes in weed infestation, fish biodiversity and detailed monitoring of the depth and quality of the water within the wetland for 1 year before, and 4 years following reinstatement of tidal flow into the Boolgooroo lagoon region of the Mungalla wetlands complex, North Queensland (Fig 1). The success of this intervention was assessed in the context of the local (Mungalla) wetland management strategy [44] which specifically targeted weed removal, and explore the broader implications of this management strategy for coastal wetland restoration.

## Methods

Ethics statement–“The fish biodiversity study was completed in accordance with the Queensland Animal Care and Protection Act 2001, and James Cook University animal ethics permit number A2178.”

### Location and climate

Mungalla Station (146^o^16’19”E, 18^o^42’55”S), is a 830 ha property located in the lower part of the Herbert River catchment south east of Ingham, North Queensland (Fig 1). Mungalla station has been run as a cattle-grazing enterprise for over a century, and since 1999 by the Nywaigi Aboriginal people, who also manage the wetland area. Until bund removal on 6th October 2013 the wetland area above the bund was palustrine with saline estuarine wetland and saltmarsh below it. Typically saltmarsh would merge into intertidal grass-sedge wetlands dominated by Bulkuru (*Eleocharis dulcis*) [46]—the common name Bulkuru is a derivative of the indigenous term “Boolgooroo” for which this wetland is named, providing a clear indicator of the wetlands pre-European state. The Boolgooroo wetland covers 60 ha of the total 160 ha Mungalla wetland complex (Fig 1), is bounded to the west by grazing lands and to the east by regrowth forest on coastal sand ridges. Mangroves and saltmarsh patches are present along the coast and to the south of the property, which is adjacent to Halifax Bay within the Great Barrier Reef lagoon. Inland, the surrounding catchment is dominated by sugar cane farms, with some areas of grazing.

The area has a wet tropical climate with highly variable seasonal and annual rainfall. The long term mean rainfall at nearby Allingham is 2060 mm and is strongly seasonal with 85% falling in the six wettest months, November to April. Temperatures are highest in December (daily average 29.1°C) and lowest in July (daily average 20.4°C), with high humidity (~63–77%) throughout the year.

Because of the highly seasonal rainfall, freshwater only enters the wetland in the wet season as direct rainfall input, runoff from the surrounding sub-catchments and overbank flow from Palm Creek, which runs along the western boundary of Mungalla station. Once the bund was removed, the upstream wetland was again connected to the coastline, with the possibility of being fed tidally with seawater from a tributary of Palm Creek (just south of Forrest Beach) and Cassady Creek (Fig 1).

### Water depth and quality measurements

Wetland water depth, temperature and electrical conductivity (EC) were monitored by loggers (CTD-Diver, Eijkelkamp Soil & Water, The Netherlands) located in five permanent positions in the wetland, beginning on 24^th^ October 2012. The locations were 450 m, 250 m and 50 m above, and, 50 m and 250 m below the bund wall (Fig 1). The above bund logger locations were all within the weed infested parts of the wetland. The loggers captured data from the bottom of the water column (10 cm above the soil surface) every 15 minutes and were downloaded as part of monthly routine maintenance visits. An additional water quality logger (Hydrolab, OTT Hydromet, Colorado, USA) was used above the bund at the 50 m location, adjacent to the Diver logger (Fig 1). The Hydrolab logger recorded values of EC, depth, pH and dissolved oxygen (DO) concentration every 30 minutes at 10 cm above the soil surface.

To examine the effect of bund removal and location (above and below the bund) on depth, EC and temperature, Two-way analysis of variance (ANOVA) was carried out. Similarly, one-way ANOVA were run to examine the effects of bund removal on pH and DO (pH and DO were not measured at the below bund location). Data for both analyses were aggregated to daily averages from the 15 and 30-minute raw data. Normality of data residuals was assessed through inspection of residual and Q-Q plots and although there was some deviation it is not problematic given sample sizes (n = 7445, CTD-Diver and n = 1218, Hydrolab), there also appeared to be some heteroscedasticity. As a result, analysis was run using a heteroscedasticity-corrected coefficient covariance matrix (hc3). ANOVA data presented are mean ± standard deviation, unless otherwise stated. Non-linear correlation was performed to assess the linear and non-linear strength of relationships between water quality measures. Time series analysis was used to detect diel variations and lags between variables. Ancillary data used in conjunction with the wetland monitoring data are daily meteorological data measured at Allingham (Australian Bureau of Meteorology (BOM) station No 032117) and tide data recorded at the Lucinda Jetty; which was then extrapolated to Forrest Beach; 1.5 km east of the wetland (4min offset). Further details of the tidal extrapolation method are given by Karim et al, [47].

### Vegetation monitoring

Vegetation monitoring and mapping in the wetland was carried out using Worldview-3 8-band satellite imagery (DigitalGlobe Inc. Longmont, CO, USA) pan sharpened to a resolution of 0.31m. Imagery was collected once each year between August and September over the Mungalla station region, then cropped to an extent around the Boolgooroo wetland (Fig 1). Classification of the imagery into major vegetation types was carried out using object-based image classification techniques [48], using nearest neighbour supervised classification, and manual classification in more difficult areas. Complexity of the wetland area required separate classification of small regions, incorporating different segmentation levels within the object-based process, which were later merged. To aid the supervised and manual classification, dominant species were recorded at 50 fixed ground truthing sites across the imagery extent each year. In addition, high-resolution aerial photography was obtained with a Go-Pro camera attached to the underside of a helicopter flying along several transects that ran parallel and across the wetland site from west to east and at an elevation of approximately 100 m. Images were geo-referenced to preselected ground control points (geo-located with a differential GPS). Major vegetation groups were clearly identified visually from the high-resolution aerial photography, which was used as further ‘ground truth’ of the World-3 imagery classification. A comparison of final classification imagery was carried out between consecutive collection dates (Aug/Sep yearly), to identify differences in the area inhabited by these major vegetation groups.

### Fish biodiversity

Prior to this project a fish survey was conducted in the wetlands using baited collapsible box traps along with cast nets, electrofishing, and visual observation at sites among the invasive vegetation [49]. For comparison another fish survey was conducted with the use of a boat-mounted electrofisher (Smith-Root 2.5 GPP generator mounted vessel) in May 2016. In this second survey, approximately one third of the wetland was surveyed over a 55 min period, around fringing vegetation and woody debris (the remaining two thirds of the wetland were too shallow for the boat to access). All native fish were identified and immediately released after capture, while pest fish species were euthanised using accepted methods. Given the differences in the sampling methods before and after bund wall removal, the data presented denotes a presence/absence of the catch records.

## Results

### Water depth and quality measurements

Two way ANOVA for measurements of depth, EC and temperature all showed statistically significant interaction between period (before and after bund removal) and location (above and below bund) *F(1*,*7744) = 223*.*87*, *p <* .*001*, *η*_*p*_^*2*^ *=* .*023*, *F(1*,*7744) = 15*.*692*, *p <* .*001*, *η*_*p*_^*2*^ *=* .*003*, and *F(1*,*7744) = 33*.*11*, *p <* .*001*, *η*_*p*_^*2*^ *=* .*004* respectively. Given the interactions simple contrasts were run that compared the difference between means for the before and after bund removal periods, above and below bund location combinations (Table 1). Additionally one way ANOVA for measurements of both DO and pH revealed a statistically significant difference between period for means of DO, *F(1*,*1217) = 271*.*36*, *p <* .*001*, *η*_*p*_^*2*^ *=* .*03I* and *pH F(1*,*1217) = 187*.*21*, *p <* .*001*, *η*_*p*_^*2*^ *=* .*027*. Contrast results comparing difference between period for mean DO and pH also appear in Table 1.

**Table 1:** Simple contrasts for water quality measurements for the above and below bund location and before and after bund removal combinations.

Dependent variable	Location	Period	Contrast	Mean difference	Std. Error	*t*	*p*
**Depth (cm)**	Above		After—Before	-21.55	0.92	23.37	<0.001
	Below		After—Before	0.60	1.15	0.52	1.000
		After	Above—Below	-14.79	0.73	-20.22	<0.001
		Before	Above—Below	7.37	1.28	5.75	<0.001
**Electrical conductivity (mS/cm)**	Above		After—Before	2.92	0.46	6.43	<0.001
	Below		After—Before	-0.39	0.57	-0.69	1.000
		After	Above—Below	-10.21	0.36	-28.32	<0.001
		Before	Above—Below	-13.52	0.63	-21.42	<0.001
**Temperature (°C)**	Above		After—Before	2.66	0.11	23.38	<0.001
	Below		After—Before	1.61	0.14	11.33	<0.001
		After	Above—Below	-0.2	0.09	-2.19	0.115
		Before	Above—Below	-1.25	0.16	-7.91	<0.001
**Dissolved oxygen (%)**	Above		After—Before	17	1.17	14.53	<0.001
**pH**	Above		After—Before	0.53	0.04	12.61	<0.001

Depth measurements:—The above bund location was deeper than the below bund location before bund removal (46.80cm ± 26.35cm vs. 39.43cm ± 26.45cm). However, following bund removal and outflow of confined water from the above bund location this relationship reversed (25.25cm ± 24.16cm vs. 40.04cm ± 31.90cm). Looking at the above and below locations individually, there was no significant change in mean water depth within Cassady creek following bund removal with mean depth remaining stable at 39.43cm ± 26.45cm before, and 40.04cm ± 31.90cm after bund removal. However, the change in water depth following bund removal was significant within the Boolgooroo wetland decreasing from a mean depth of 46.80cm ± 26.35cm to 25.25cm ± 24.16cm.

**Fig 2:**
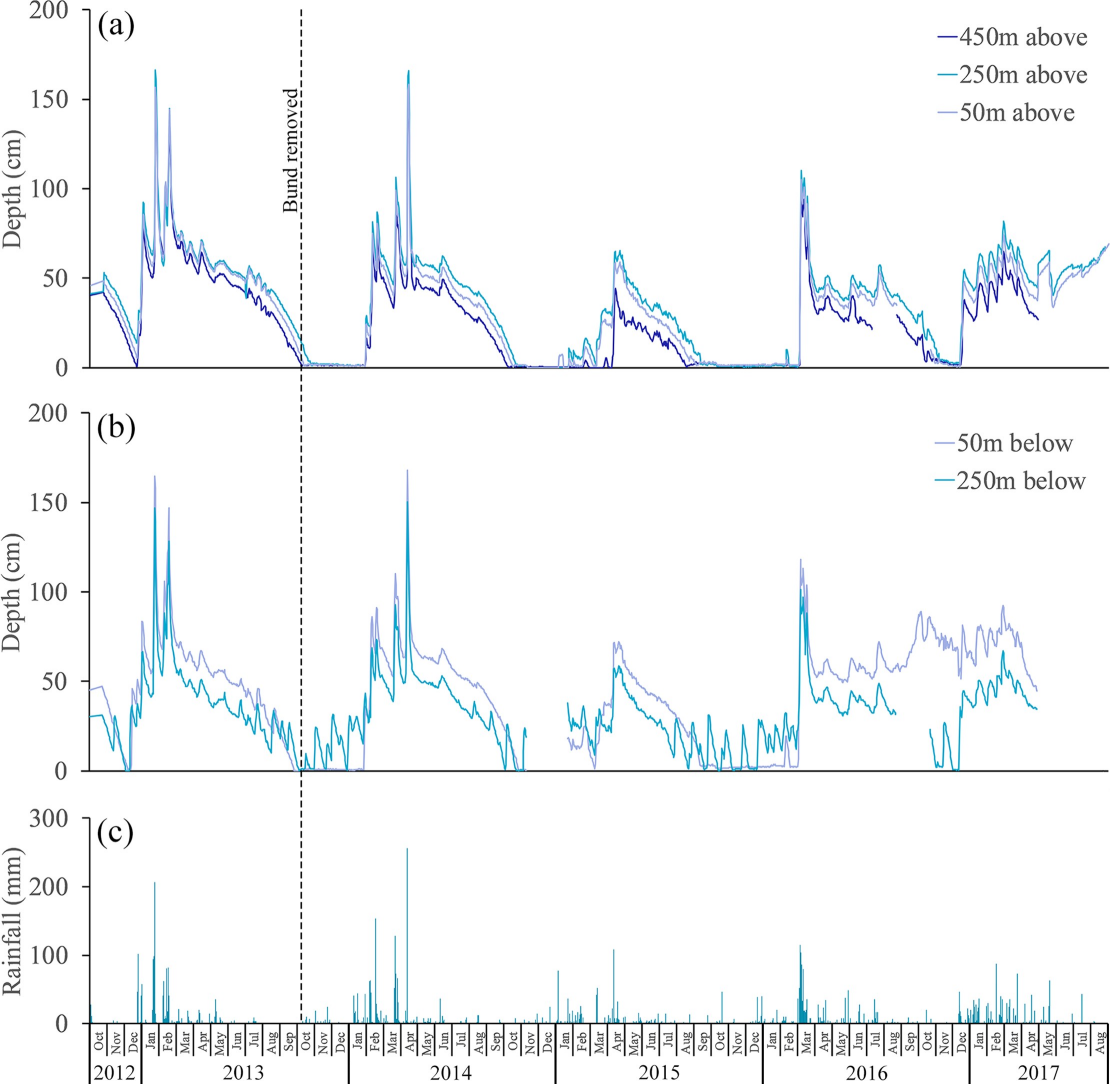
Changes in depth above and below the bund location and daily rainfall—October 2012 to August 2017. Logger distances above the bund (a) are 450 m, 250 m and 50 m and below (b) are 50 m and 250 m. The bund was removed on the 6^th^ October 2013. Daily rainfall (c) was derived from the nearby Bureau of Meteorology station at Allingham approximately 2.5 km distant.

During 2012 wetland depth at all locations declined steadily with only 0 to 20 cm of water depth above the bund and even less (0 to 10 cm) below the bund (Fig 2A and 2B) by the middle of December 2012. Subsequent rainfall (Fig 2C) increased the wetland depth, which reached a maximum of ~170 cm (above and below the bund) at the end of January 2013.

**Fig 3:**
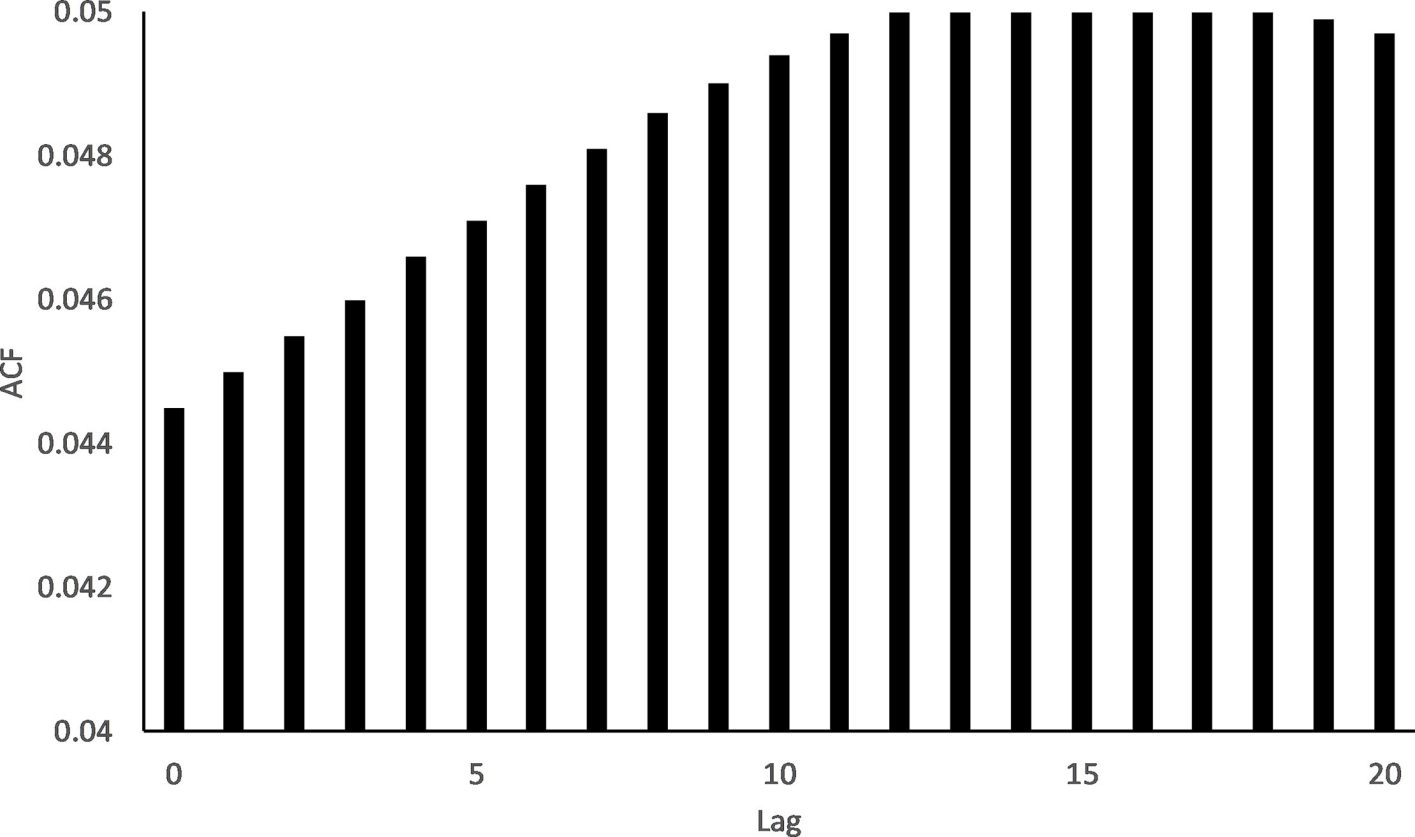
Cross-correlation between tide height and depth measured at the 250m below bund location. Maximum correlation occurs at a lag of 12 to 17 which is equivalent to a 3 to 4.25 hr delay between tides experienced at the coast and measured changes in depth at the 250m below bund location– 15 min data records. 95% CI has been omitted from the graph falling at ± 0.05.

After February 2013, water levels dropped rapidly, and then more slowly as drainage from the wetland slowed. Smaller rainfall inputs to the wetland in March, April and May 2013 resulted in only modest increases in water depth (~ 10 cm). After this time water depth again dropped steadily, approaching zero in October 2013 just before bund removal. During this same period there were multiple occasions when depth increased, at approximately monthly intervals at the below bund locations (Fig 2B) with no corresponding increase in depth above the bund (Fig 2A); therefore it would appear that the depth increases below the bund were due to tidal pulses alone indicating that there was no tidal penetration beyond the bund before its removal. Further investigation, using cross-correlation between tide height at the coast and depth at 250m below the bund, revealed that there is a lag of 3 to 4.25 hrs between coastal inundation and change in depth at the below bund location (Fig 3).

Following bund removal (6^th^ October 2013), re-establishment of connectivity to Cassady creek was immediately evident with slow freshwater outflow from Boolgooroo lagoon to reach an equilibrium depth of ~10 cm lower than the bunded state at that time. The 2013/14 wet season (November to April inclusive) had just below average wet season rainfall (1443 mm) that did not start until late-January 2014. As a result, water depths at most of the locations remained close to zero from the end of October 2013 to mid-February 2014. In contrast, depths at the 250 m below location continued to rise at monthly intervals, corresponding to the high tides at the nearby coast. None of the tides in November and December 2013 reached further than the 250 m below location. However, very large tides at the end of January 2014, culminating with 4.3 m, reached further inland inundating both the 250 and 50 m below locations as well all three monitored locations within the Boolgooroo wetland increasing wetland depth to 25cm at 50m above the bund location and 16cm at the 450m above bund location. Changepoint analysis of wetland depth and tide height during periods where inundation occurred before the influence of rainfall (2014,2015,2016) indicate that 3.63m (SE = 0.29, 95% CI [2.89,4.03]) is the lower limit of tide height that will increase water depth within the wetland (measured at the 50m above bund location). The 4.3 m tide experienced at that time is within the 95^th^ percentile for the region and a good indicator of maximum tidal inflow that could be expected within the wetland. The second wet season after bund removal (2014/15) had only 47% of average wet season rainfall (751 mm) leading to relatively low wetland depths (maximum ~ 70 cms) in this season (Fig 2A) falling to very low levels in August 2015, continuing to dry out until December of 2016 when tidal flow marginally affected the lower wetland before drying out again until February 2016. The 2015/16 wet season started late with the majority of rainfall falling in March 2016, continuing until July 2016. As a result, mean wetland depth remained high, eventually falling to close to zero for a very short period in December 2016, with rainfall then maintaining water levels at >50cm until the end of the study in August 2017.

**Fig 4:**
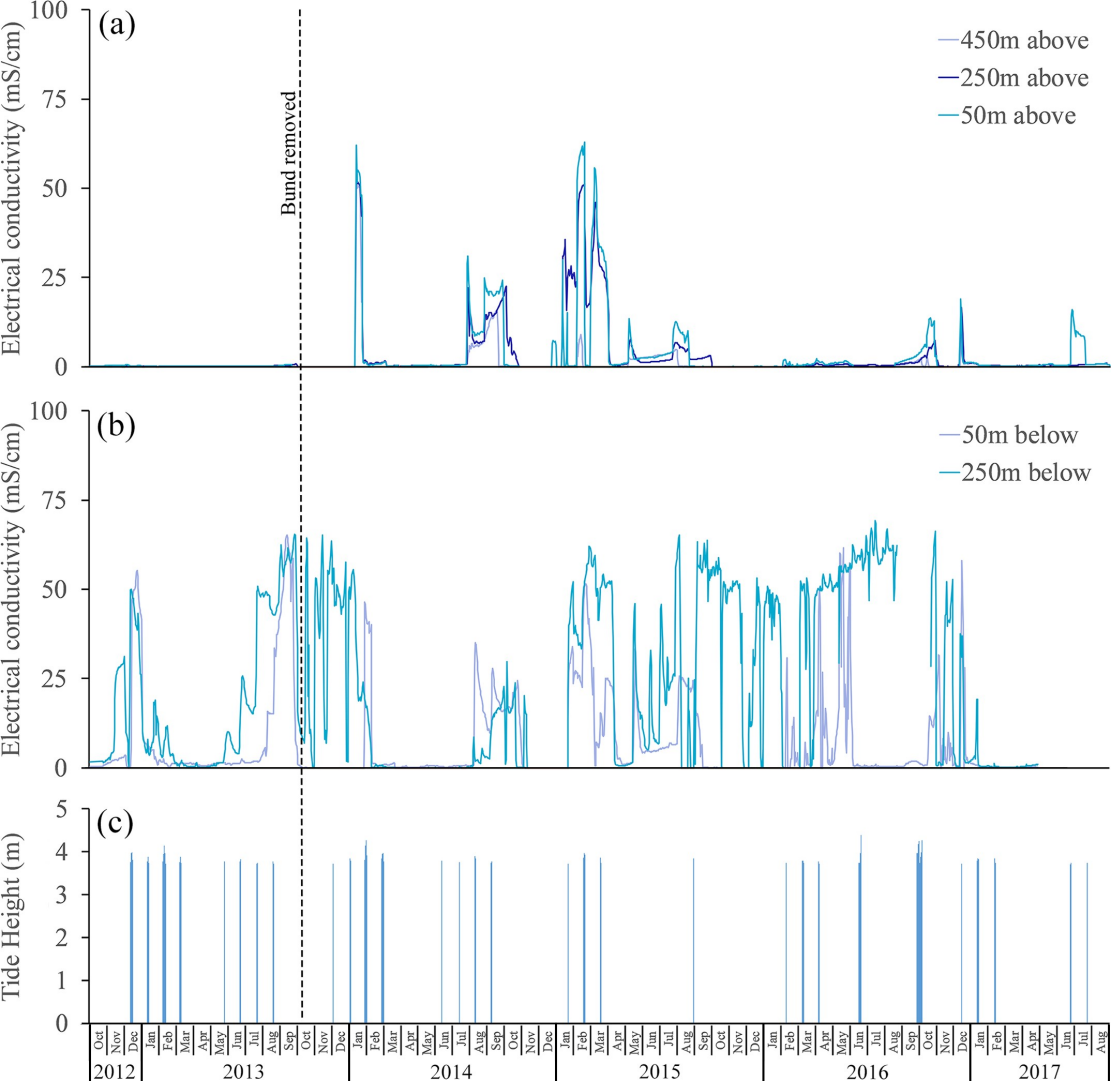
Changes in salinity above and below the bund location and tides > = 3.6 m—October 2012 to August 2017. Logger distances above the bund are 450 m, 250 m and 50 m and below are 50 m and 250 m. The bund was removed on the 6th October 2013. High tides (> = 3.6 m) are for Forrest Beach.

Electrical conductivity (EC) measurements:—Prior to bund removal the Boolgooroo wetland remained freshwater, 0.29mS/cm ± 0.11mS/cm, compared to the brackish waters of Cassady creek, 13.82mS/cm ± 19.35mS/cm. Following bund removal, the mean EC at the above bund location, whilst becoming brackish, remained significantly lower than the below bund location (3.22mS/cm ± 8.77mS/cm vs. 13.42mS/cm ± 19.39mS/cm). Looking at the above and below bund locations individually, there was no significant change in EC at the below bund location following bund removal with mean EC remaining stable in Cassady creek below the Boolgooroo wetland at 13.82mS/cm ± 19.35mS/cm before and 13.42mS/cm ± 19.39mS/cm after bund removal. However, the change in EC following bund removal (due to sea water ingress) was significant within the Boolgooroo wetland itself increasing from a mean EC of 0.29mS/cm ± 0.11mS/cm to 3.22mS/cm ± 8.77mS/cm. In January 2014 the wetland experienced the first single tides over 3.6 m which did not affect the wetland above the bund location. These were followed by 5 tides over 3.6m at the end of the month (Fig 4C).

**Fig 5:**
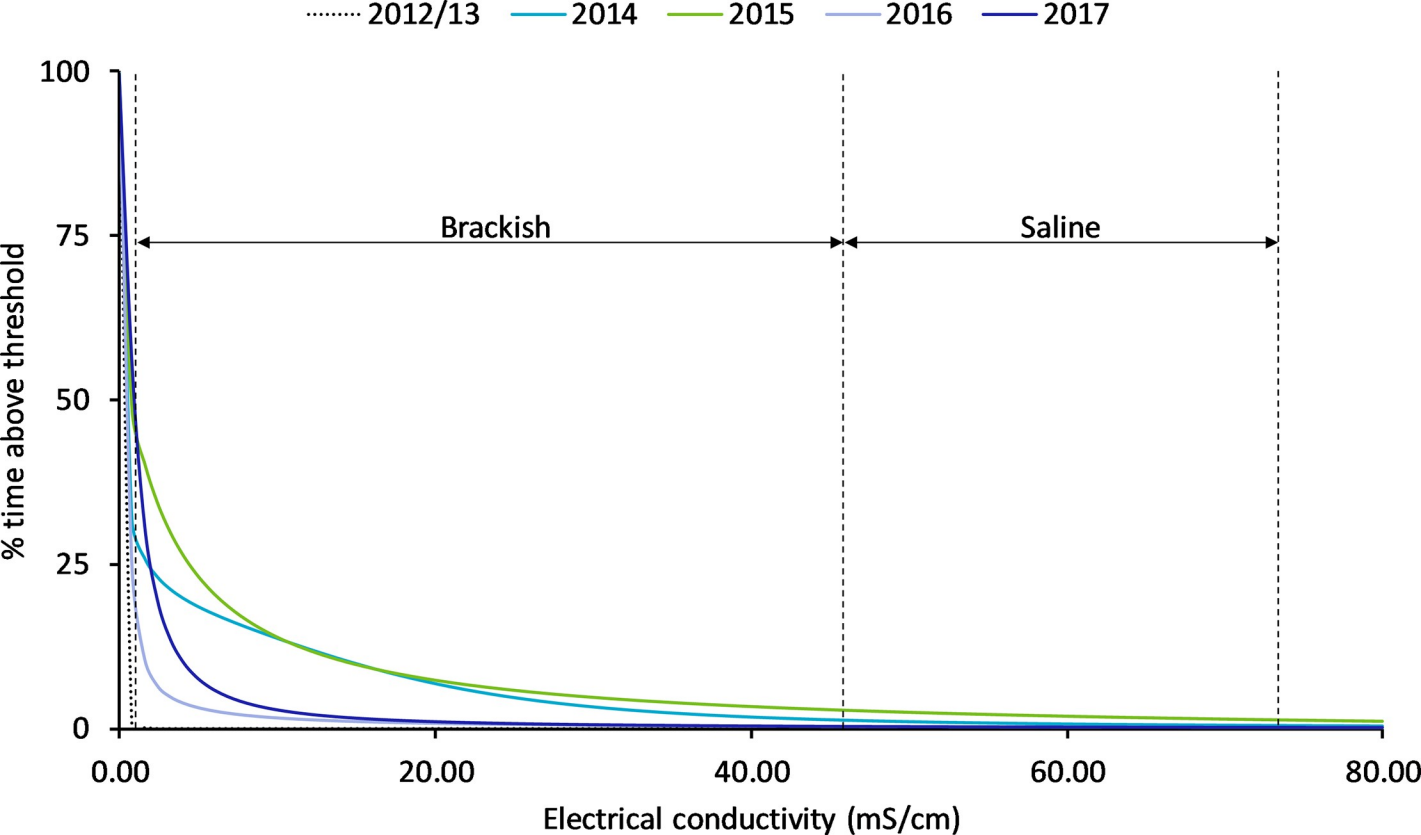
The percentage of time EC exceeded any given EC threshold during pre and post bund removal years within the Boolgooroo wetland. Horizontal dashed lines show boundaries between fresh, brackish, saline and briny water from left to right.

These tides were unusually high due to a severe tropical low in the Coral Sea at that time. As a result, the wetland became influenced by tidal ingress (> 46 mS cm^-1^) and remained so before rainfall again suppressed conditions (Fig 4A and 4C), with conductivity levels becoming brackish (> 1 < 46 mS cm^-1^) before returning to freshwater (< 1 mS cm^-1^). In 2014 there were 7 tidal periods (most with 3 consecutive tides over 3.7m) that affected the wetland through increased residence times of brackish and saline water (Fig 5). The following year (2015) saw 4 tidal periods over 3.7 m with 3 periods being recorded at the 450 m above bund location. 2015 was the driest year on record for the region, which accounts for the differences in tidal inundation residence times (Fig 5), as wetland depth and associated freshwater outflow did not restrict tidal inflow to the same degree as the previous year.

**Fig 6:**
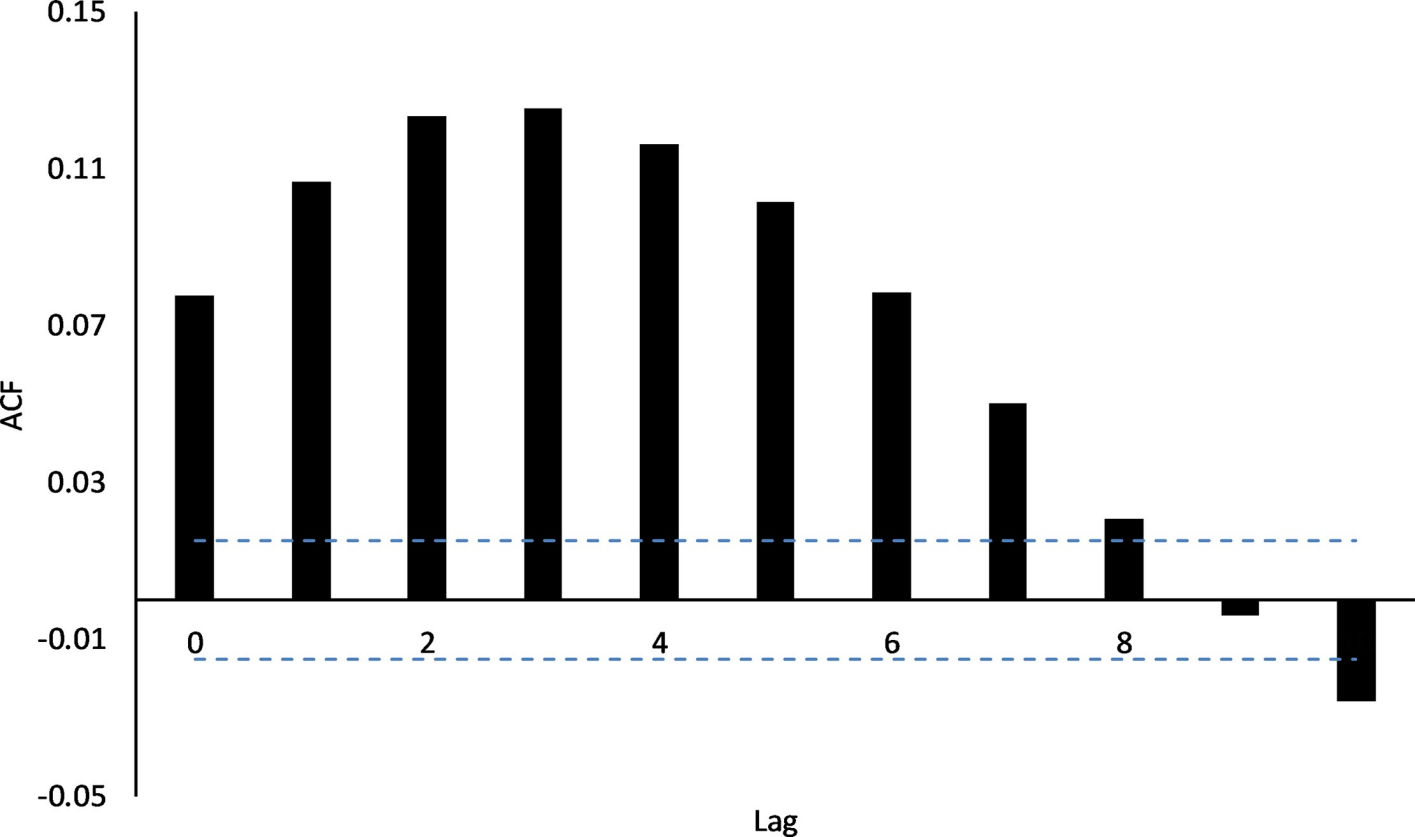
Cross-correlation between tide height and EC measured at the 50m above bund location. Maximum correlation occurs at a lag of 3 which is equivalent to a 3-day delay between tides experienced at the coast and measured changes in EC.

In addition, lack of freshwater flows in the 2014/15 season also led to less dilution of the seawater in the wetland with evaporation processes reducing wetland depth markedly and contributing to particularly high (>55mS/cm) and prolonged salinity levels in the wetland (Figs 2A and 4A). Notably there was a single high tide of ~3.7 m in February of 2016 which did not enter the wetland (Fig 4A and 4C) with a similar occurrence in early January of 2014. On further investigation cross-correlation of tide height and EC (using daily aggregated data from the 50m above bund site) revealed a maximum correlation at a lag of 3 days (Fig 6) giving an indication that maximum increases in EC will only occur when there are 2–3 consecutive tides.

**Fig 7:**
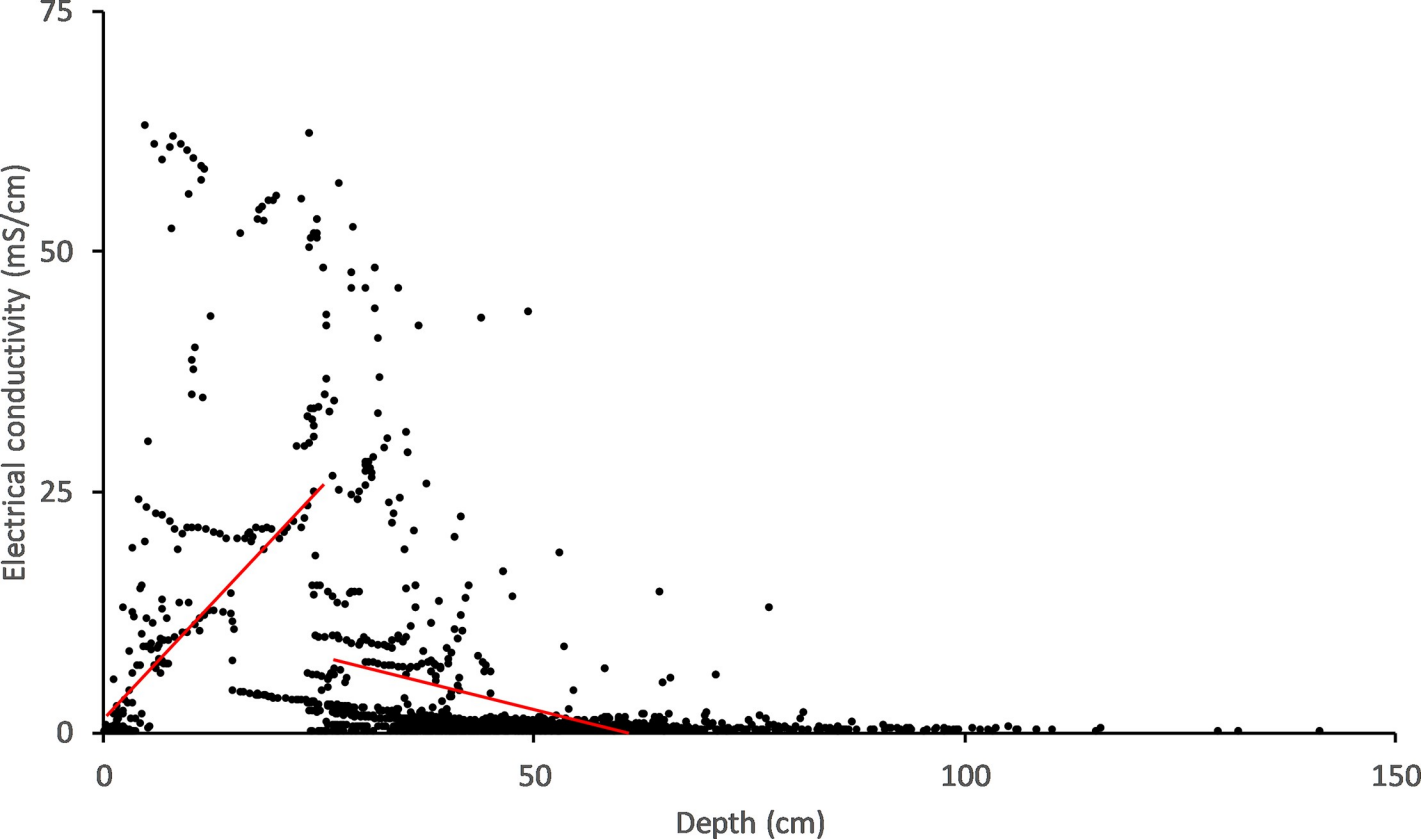
Piecewise visualisation of non-linear correlation between depth and EC. Red lines represent linear correlation segments from within the non-linear correlation. Here we can see increases in EC with Depth and decreases in EC with depth associated with tidal ingress and rainfall respectively.

Further investigation using non-linear correlation between depth and EC reveals a moderate but significant relationship (r = 0.39, P < 0.01). Piecewise correlations for the above analysis are visualised in Fig 7, showing increases in EC with increases in depth representing tidal inundation, along with decreases in EC with increased depth due to rainfall.

Changepoint analysis of this dataset (a subset of the 50m above bund location including only low rainfall periods) indicates that EC stops increasing at a wetland depth of 36cm (SE = 3.79, 95% CI [28.31,43.15). Therefore, not only are several high tides required to increase wetland EC, it will only occur when wetland depth is below ~40cm, or outflow is non-existent or very low as in January of 2015. There was a large amount of rainfall during March of 2016 with constant rainfall input until July. The wetland remained as freshwater until early October with tidal water reaching upstream areas of the bund wall in December resulting in only small conductivity peaks (Fig 4A). In the final 7 months of the study (2017) 3 tides affected the wetland, but only to the above 50 m location (Fig 4A) with minor impact.

**Fig 8:**
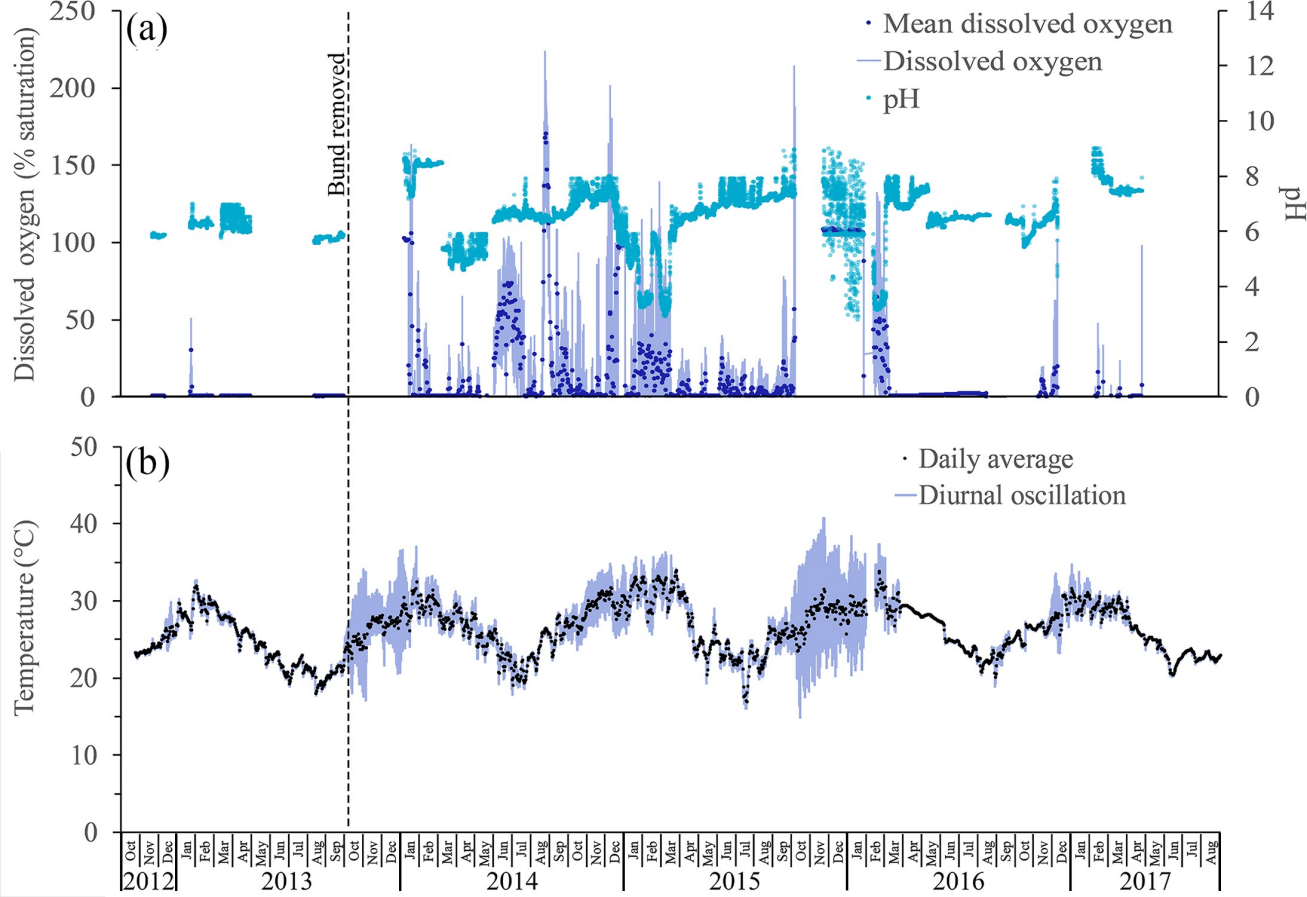
Seasonal changes in dissolved oxygen, pH and temperature. Daily average dissolved oxygen (% saturation) and pH were recorded by the Hydrolab logger at the above 50 m location (a). Daily average water temperatures shown are as measured 250 m above the bund location (b). Diurnal oscillations in dissolved oxygen (30-minute intervals) and water temperature (15-minute intervals) are also shown (a), (b).

Temperature:—Prior to bund removal the above bund location was significantly cooler than the below bund location, (24.50°C ± 3.57°C vs. 25.75°C ± 3.19°C). However following bund removal, the mean temperature for the above and below bund location increased significantly, becoming uniform (27.16°C ± 3.24°C vs. 27.36°C ± 3.42°C) (Table 1). Water temperature in the wetland following bund removal varied seasonally, being coolest in June/July (mean = 22.5°C ± 1.7°C) and warmest in January/February (mean = 30.7°C ± 1.6°C) (Fig 8B).

**Fig 9:**
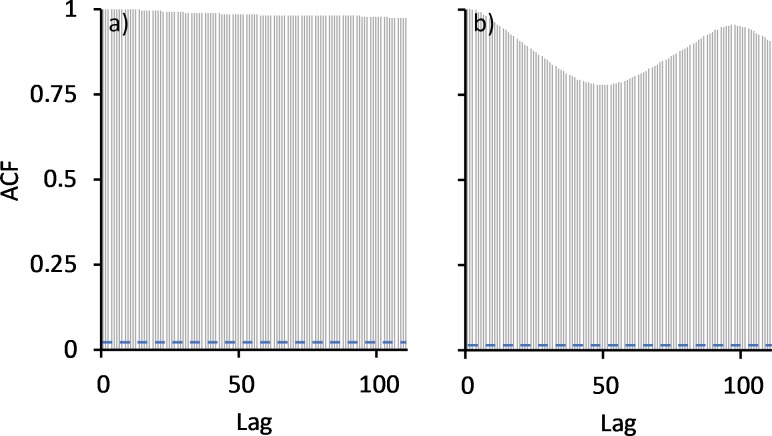
**Autocorrelation of temperature at the above bund locations, a) before and b) after bund removal.** Lag distance here is 15 min with a 24 hr period represented by a lag of 96.

Water temperatures at the three locations above the bund were very similar with the highest temperatures being recorded in January/February 2015, averaging 31°C ± 1.7°C above the bund location, compared to 29.5°C ± 1.2°C and 29°C ± 1.2°C in the two previous summer seasons, and to 30.5°C ± 0.8°C and 29.4°C ± 0.8°C in the following two seasons. The higher temperatures in 2015 coincided with the low rainfall received that wet season, leaving the wetland very shallow, averaging only 11 cm above the bund location in January and February (Fig 2A). Autocorrelation analysis reveals that prior to bund removal water temperature did not exhibit diel variation (Fig 9A).

**Fig 10:**
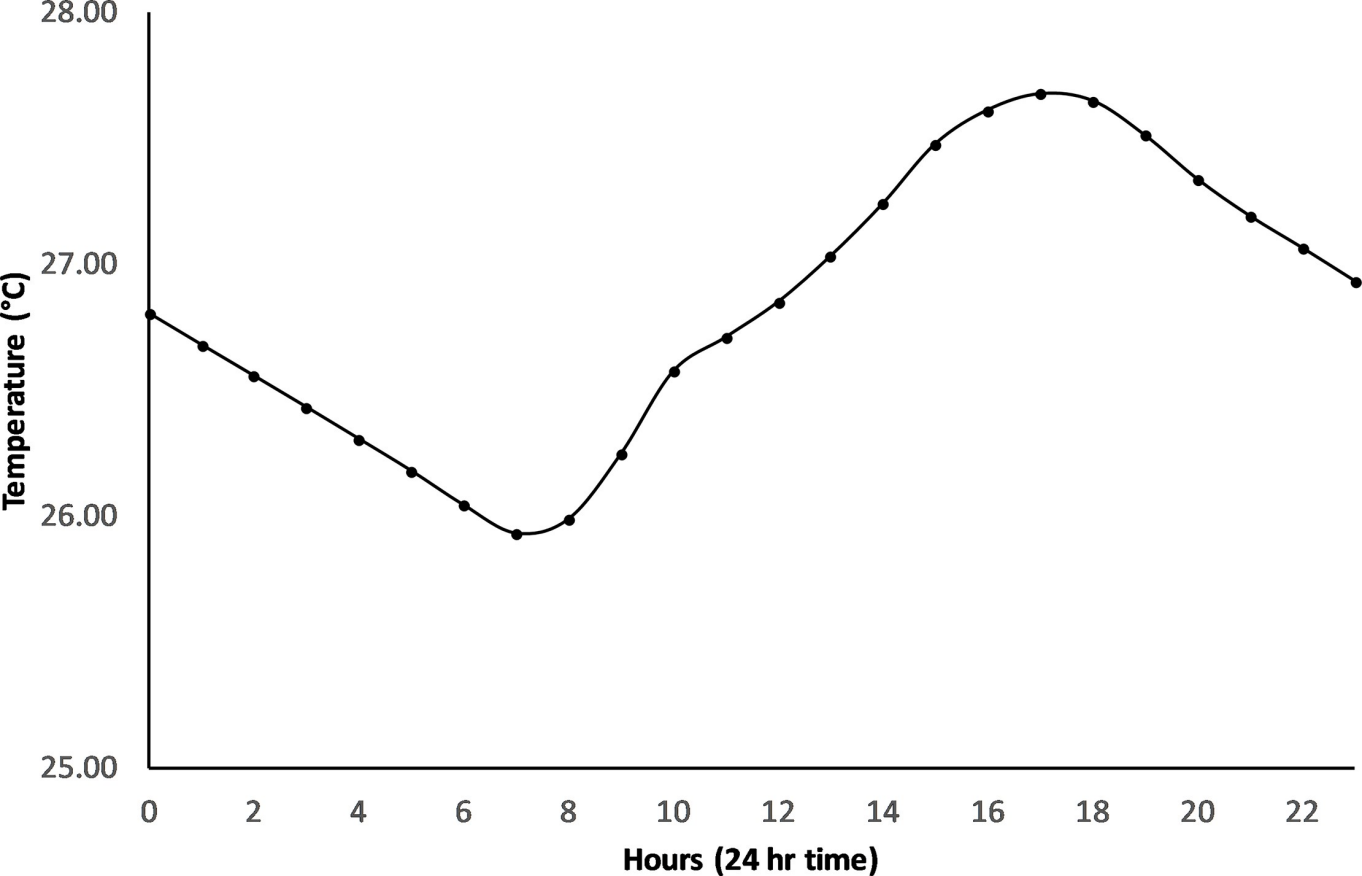
Mean hourly temperature for the post bund period– 2014 to 2017.

However, this changed following bund removal (Fig 9B) with temperature reaching a maximum at 5pm falling to a minimum at 7am (Fig 10).

**Fig 11:**
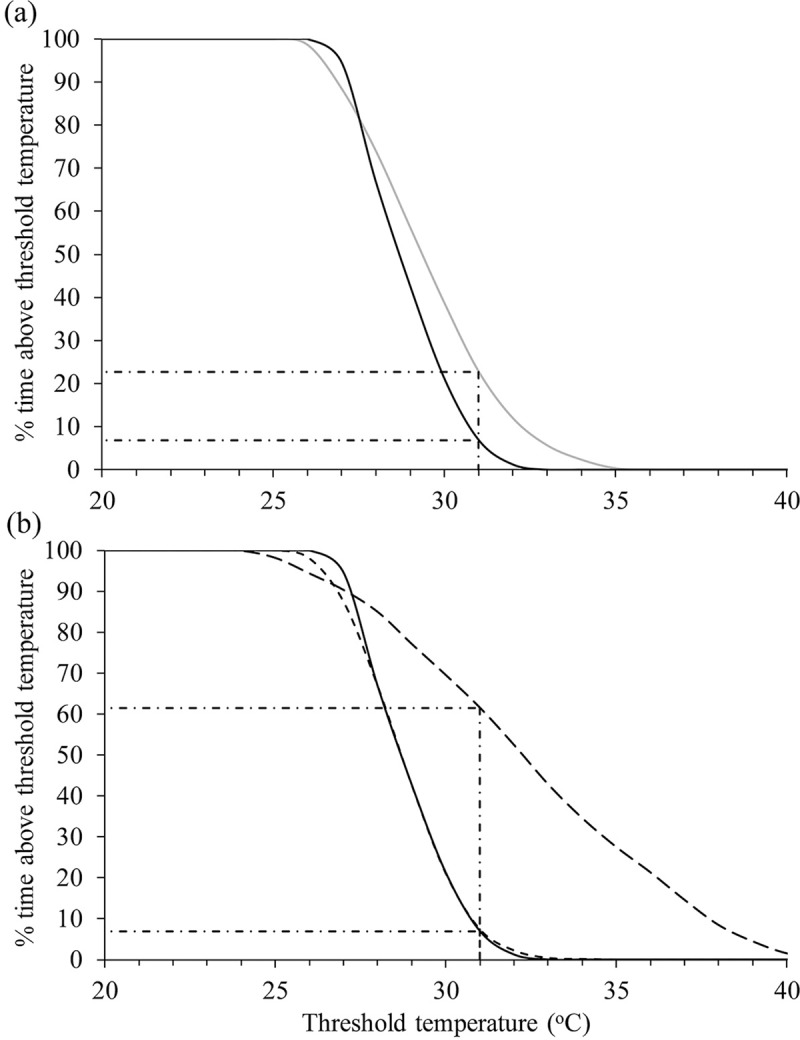
The percentage of time water temperature exceeded any given temperature threshold during the warmest months (January, February and March) in the Mungalla wetland (Boolgooroo). (a) compares the temperatures 250 m above (black) and 250 m below (grey) the bund before it was removed. (b) compares the temperatures 250 m above the bund before (black) and after (2014; short-dashed black; 2015; long-dashed black) it was removed. The exceedance of threshold T_pref_ = 31°C is also shown.

There were notable periods when the diel oscillations increased to > 10 degrees; for example, during the period of October 2013 to January 2014 and October 2015 to January 2016 (Fig 8B). Large oscillations in temperature coincided with periods of shallow wetland depths. The thermal regimes 250 m above and 250 m below the bund are compared in (Fig 11). These plots show how often water temperature exceeded a given temperature threshold and are compiled from all 15-minute recordings made from the warmest time of the year in January, February and March (JFM) 2013 (before bund removal) and the same three months in 2014 and the driest year 2015 (after bund removal).

Water above the bund was greater than 26°C during JFM 2013, but rarely exceeded 32°C (Fig 11A). Water was warmer below the bund during this period, reaching 35°C. The exceedance of the preferred temperature threshold for some tropical freshwater fish (see [50]), T_*pref*_ = 31°C, was much higher below the bund (23% of the time) compared with above the bund (6.9% of the time). Comparison of 2013 and 2014 thermal frequency curves (Fig 11B) shows that bund removal *per se* did not markedly affect the wetland thermal regime. However, water temperature was very dependent on (shallow) wetland depths, which were mainly determined by the timing and amount of wet season rainfall. This was the situation in the 2014/15 season where the low rainfall led to shallow wetland depths (Fig 2A). As a result, diel temperature oscillations were very high (Fig 8B) and led to prolonged periods of high temperature, as shown by the temperature frequency curve for JFM 2015 (Fig 11B). For example, the exceedance of the temperature threshold T_*pref*_ = 31°C, was 61% in JFM 2015, compared with 7.3% and 6.9% in 2014 and 2013 respectively. Temperatures never exceeded 34°C in 2013 and 2104, but this temperature was exceeded 34% of the time in 2015, and even approached 40°C at times.

**Fig 12:**
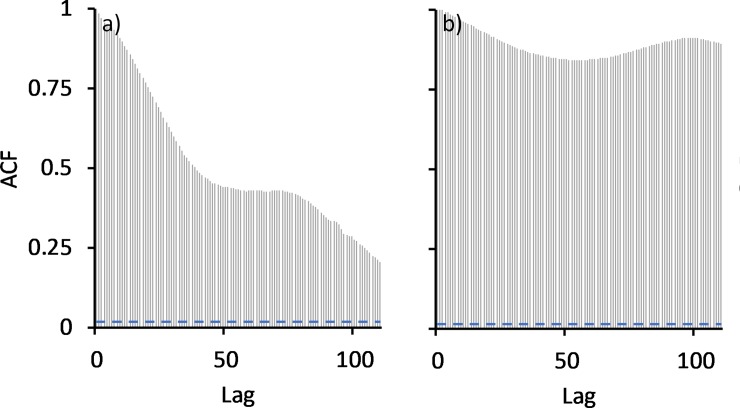
**Autocorrelation of DO at the above bund locations, a) before and b) after bund removal.** Lag distance here is 15 min with a 24-hr period represented by a lag of 96.

**Fig 13:**
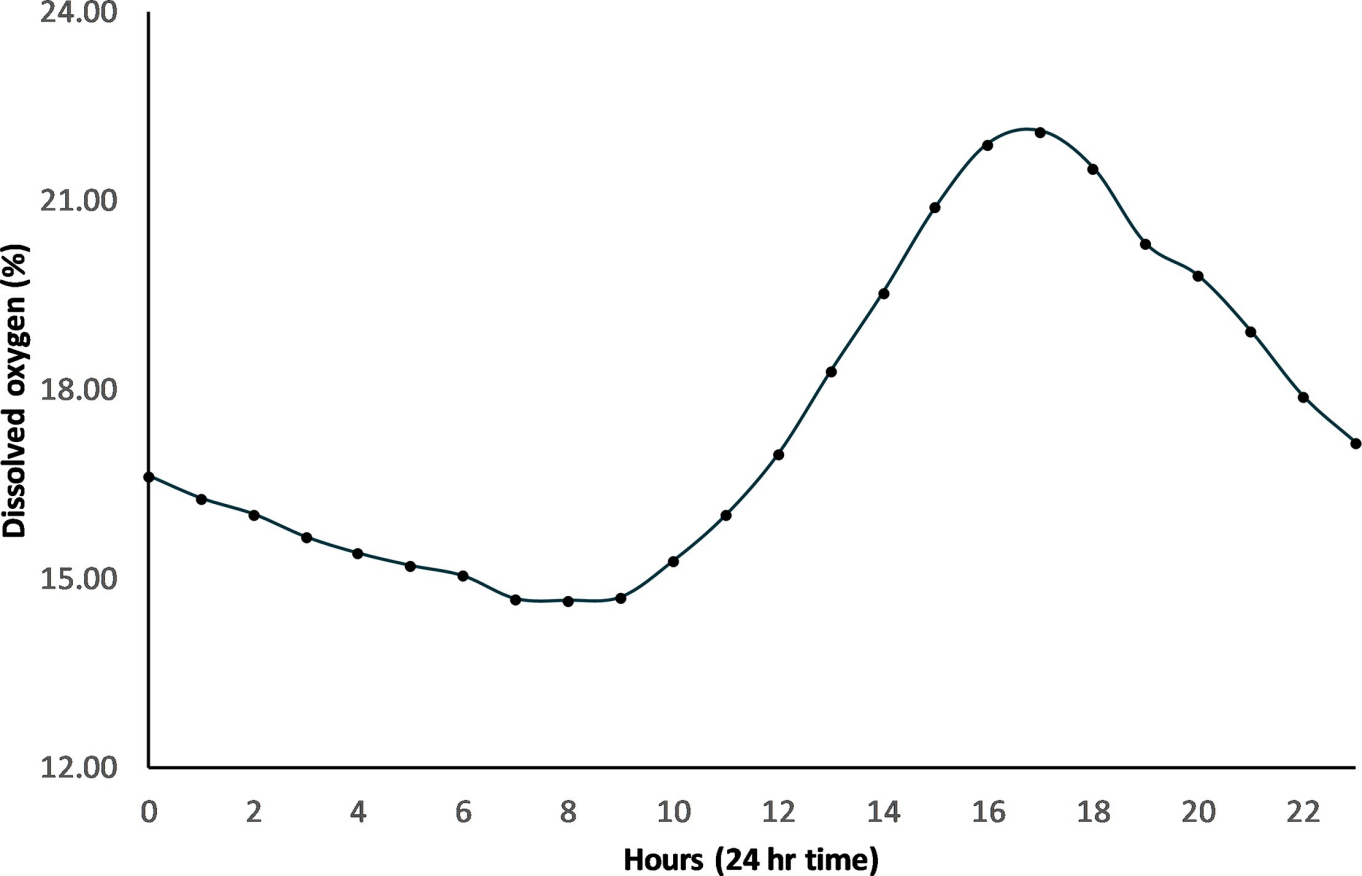
Mean hourly DO for the post bund period– 2014 to 2017.

Dissolved oxygen % (DO):—Dissolved oxygen saturation (recorded 10 cm above the bottom of the wetland) was consistently close to zero before the bund was removed (mean = 0.72% ± 2.45%) (Fig 8A) with no diel variation in DO saturation evident (Fig 12A). However, in the first wet season after bund removal, DO improved following the first series of seawater pulses that entered the wetland in January and February 2014 (Fig 4A), reaching ~ 100% saturation (Fig 8A). Diel variation in DO saturation became evident at this time (Fig 12B), continuing across the post bund period, reaching a maximum late in the afternoon (Fig 13).

**Fig 14:**
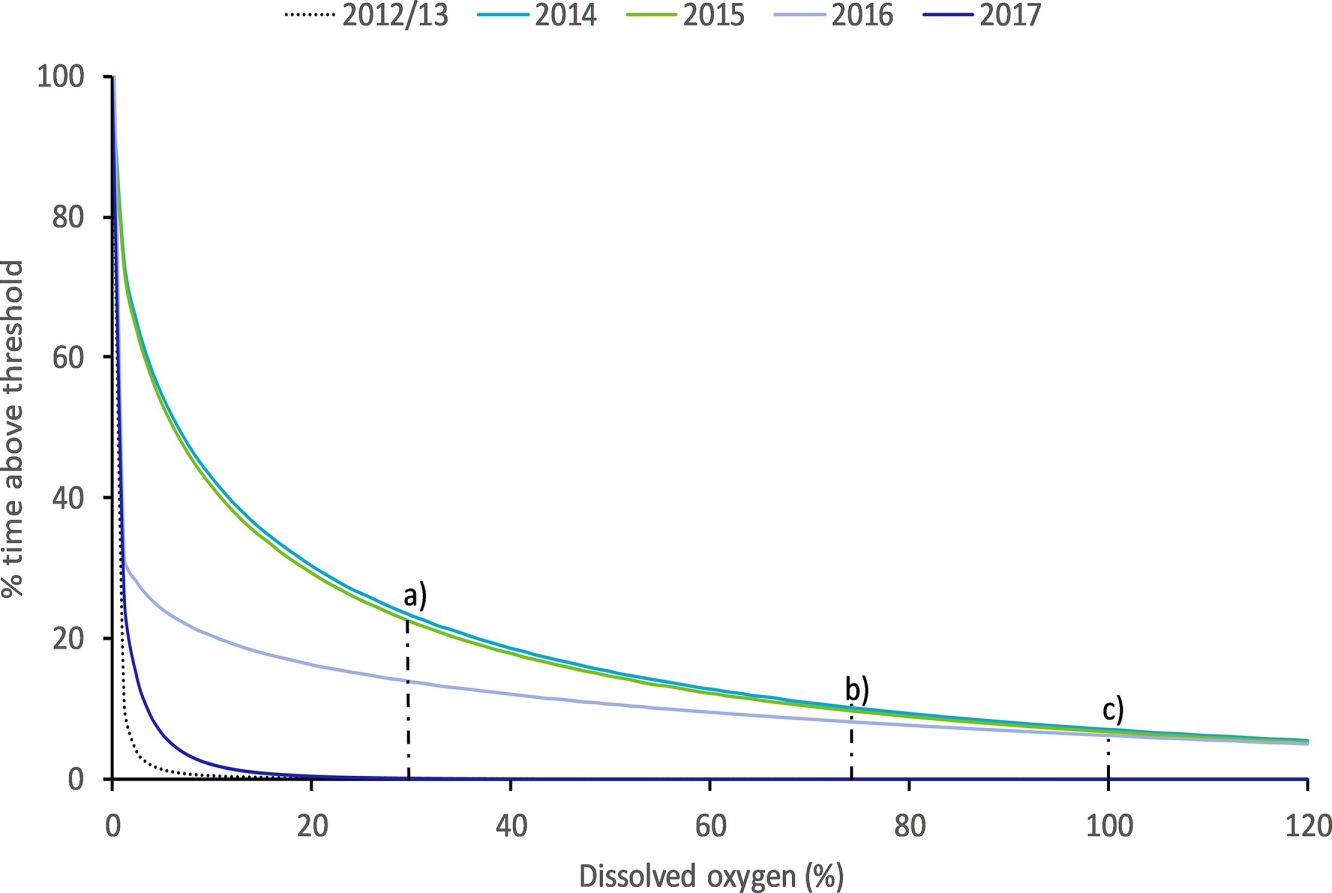
The percentage of time DO exceed any given DO threshold in the Boolgooroo wetland. Vertical dashed lines represent critical values of DO affecting fish species, a) acute trigger value, b) chronic trigger value and b) to c) optimal DO for fish species. From dissolved oxygen guidelines for freshwater habitats of Northern Australia [51].

DO declined again for several weeks after this until the freshwater pulses in March and April 2014 again improved DO; however, initially there were relatively few days when DO reached 100% saturation. By June and July 2014 DO improved further, approaching 100% saturation on many days. As wetland depth dropped below 40 cm in August and September 2014, the seawater pulses that could now enter the wetland continued to sustain reasonably high DO with 7 days exceeding a daily average DO of 100% about two weeks after the seawater ingress in August 2014. Despite the low water levels in January and February 2015, DO concentrations reached 80 to 100% on many days, however much lower DO was recorded during the next few months until an increase in August following a seawater pulse. There were few increases in DO in 2016 with DO generally remaining very low compared to the two previous years. 2017 levels approached pre-bund removal periods (Fig 14) remaining below acute DO levels [51].

Some spikes did occur after tidal intrusion in February of 2016 directly following drying out of the wetland, and again in October to November following tidal intrusion. 2017 experienced very few short-lived increases in DO between February and April of that year due to rainfall, with DO remaining close to zero. Generally, while DO has improved following bund removal it has remained low with only 8–10% of the time between 2014 and 2016 being above the critical DO saturation level for maintaining healthy fish species in this region.

**Fig 15:**
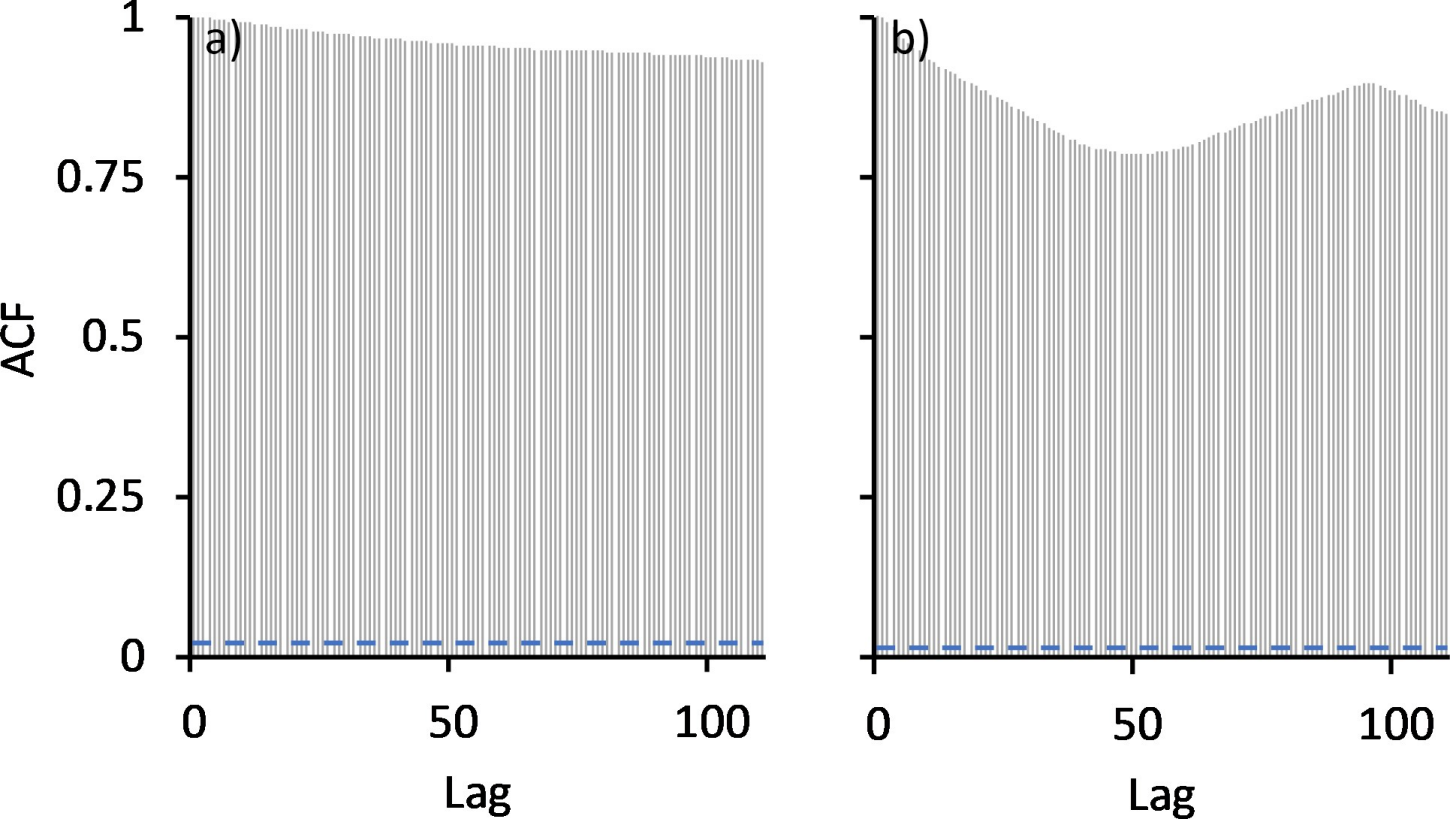
**Autocorrelation of pH at the above bund locations, a) before and b) after bund removal.** Lag distance here is 15 min with a 24-hr period represented by a lag of 96.

pH:—pH recorded above the bund location was more acidic before bund removal compared to post bund removal levels (Fig 5A) (6.08 ± 0.21 vs. 6.60 ± 1.13). As with temperature and DO, pH exhibited diel variation following bund removal but not before (Fig 15).

**Fig 16:**
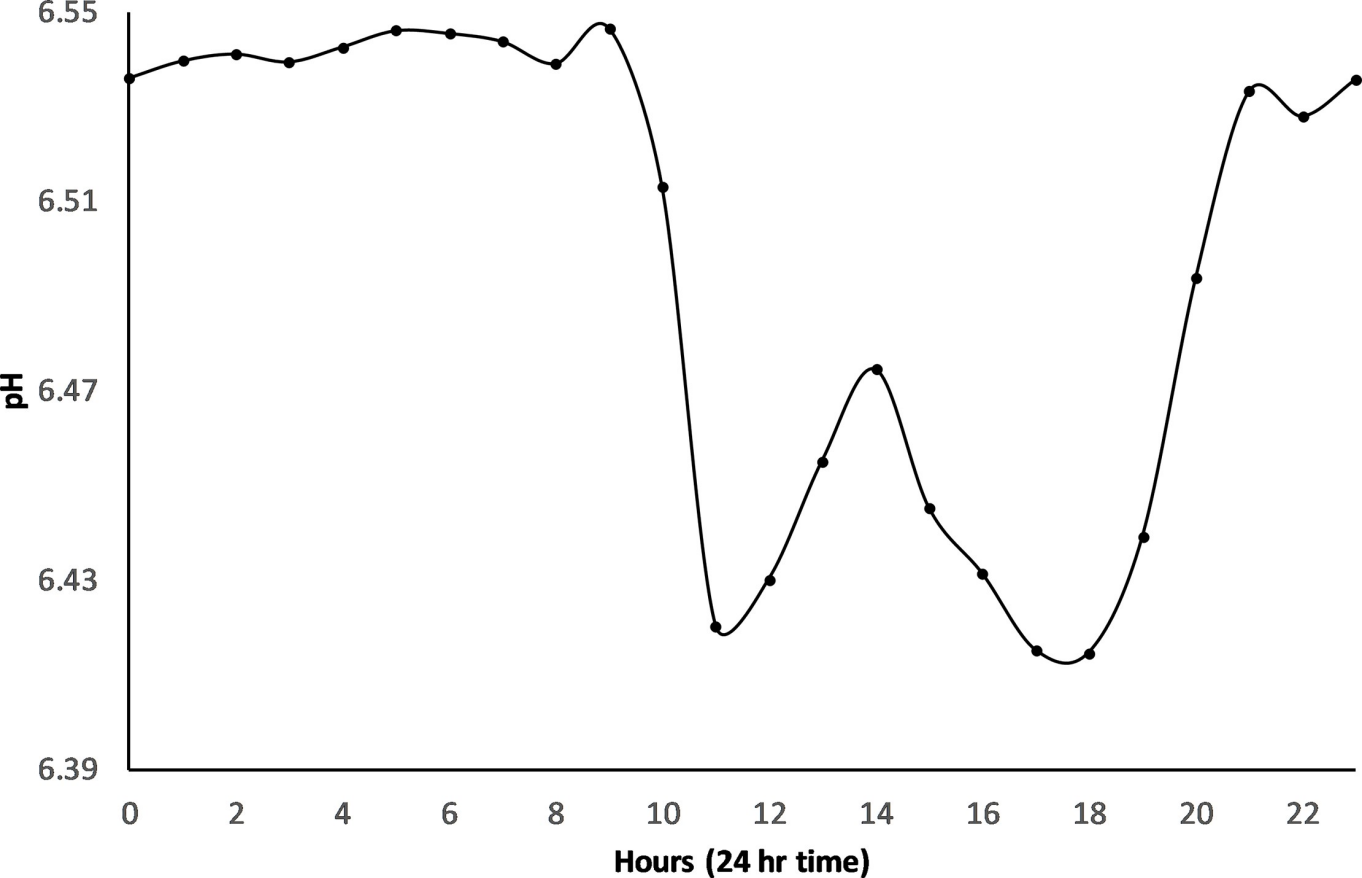
Mean hourly pH for the post bund period– 2014 to 2017.

Diel variation of pH did not fit the same pattern as with DO and temperature, instead being highest overnight (9pm to 9am) and falling during the day with lowest values of pH occurring at 11 am and 6pm. There is an increase in pH occurring between the two low points during the day reaching a maximum at around 2pm (Fig 16). This increase in pH does not match overnight levels however.

Occasions were recorded in early 2015 and again at the end of 2015 into 2016 when pH became acidic (< 4). These periods followed drying out of the wetland and reflect leaching of sulphuric acid following oxidation of acid sulphate soils within the upper Boolgooroo. Low pH levels were short lived however and pH was restored to within ranges expected in wetlands in the region [52] following buffering by tidal intrusion or rainfall. There were also a number of periods when pH rose above 8 following tidal intrusion (regional coastal sea water has a pH of 8.1) (Fig 8A).

### Vegetation monitoring

**Fig 17:**
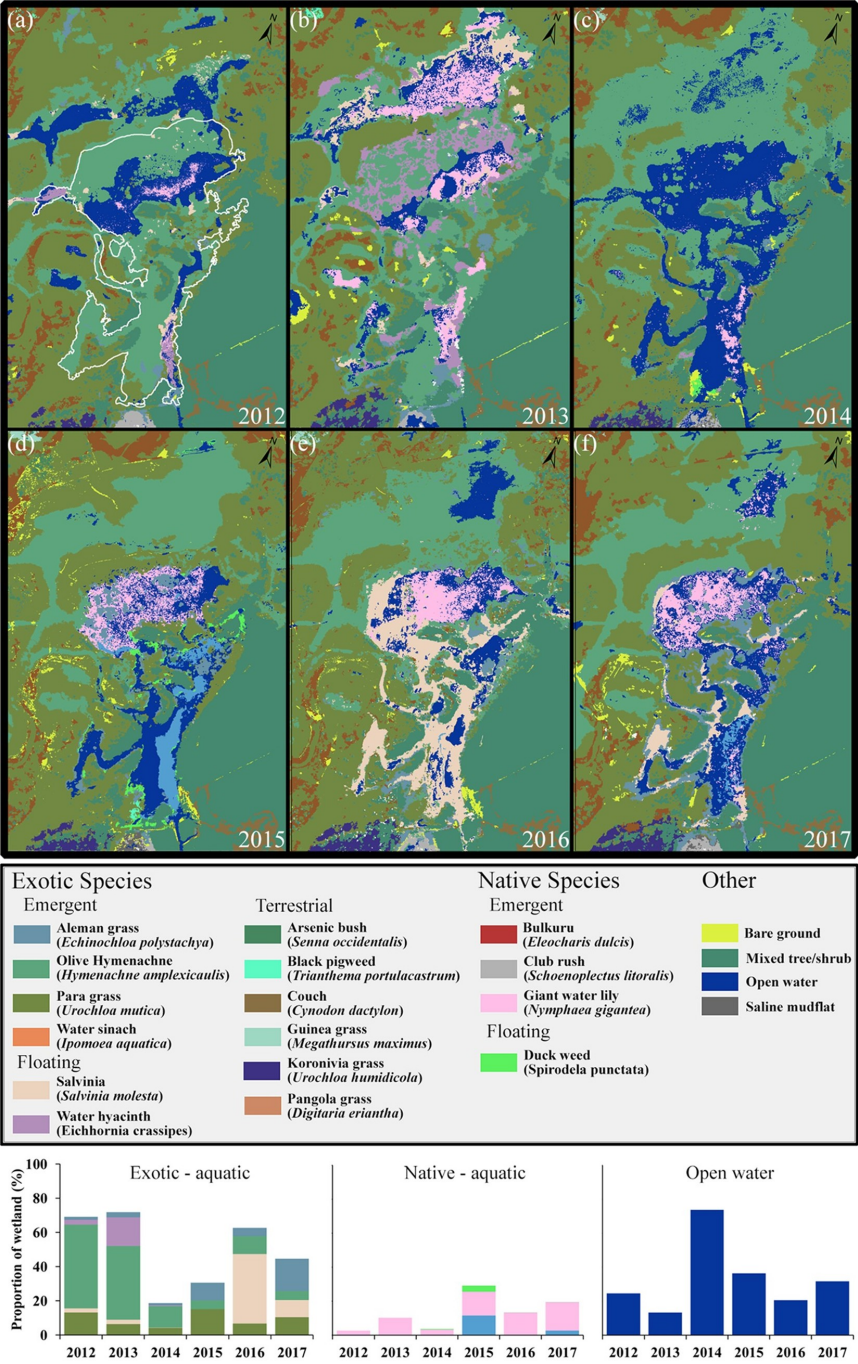
Changes in vegetation from 2012 to 2017. The white outline in (a) represents the Boolgooroo region on which change analysis was based. Charts on the bottom of the figure represent changes in exotic/native vegetation and open water (colours correspond to the classification key).

Prior to bund removal (2012/13), with no changes in EC above the bund, over two thirds of the area was inhabited with ponded pastures and exotic floating vegetation. A small percentage was inhabited by *Nymphaea gigantea*: a native water lily during 2012 which increased into open waters during 2013 (Table 2), (Fig 17A and 17B). Over half of the area was infested with weeds of national significance (WONS) (Table 2), dominated by *Hymenachne amplexicaulis* (olive hymenachne) and *Eichhornia crassipes (*water hyacinth*)*. On average open water made up less than one fifth of the Boolgooroo area (Table 2).

**Table 2:** Area inhabited by vegetation groups (%).

	Survey Year					
Exotic aquatic plants	2012	2013	2014	2015	2016	2017
Aleman grass† *(Echinochloa polystachya*)	1.85	3.12	1.18	10.26	4.87	18.93
Olive hymenachne*† (*Hymenachne amplexicaulis*)	48.94	43.04	12.61	5.11	10.51	5.23
Para grass† (*Urochloa mutica*)	13.26	6.45	4.24	15.16	6.83	10.42
Salvinia* (*Salvinia molesta*)	2.41	2.61	0.04	0.00	40.53	10.12
Water hyacinth* (*Eichhornia crassipes*)	2.68	16.72	0.55	0.03	0.00	0.00
**Native aquatic plants**						
Bulkuru (*Eleocharis dulcis*)	0.00	0.00	0.00	11.46	0.26	2.59
Club rush (*Schoenoplectus litoralis*)	0.00	0.00	0.00	0.00	0.00	0.10
Duck weed *(Spirodela punctata)*	0.00	0.00	0.31	3.57	0.00	0.00
Water lilily (*Nymphaea gigantea*)	2.66	10.16	3.31	14.07	12.91	17.54
**Other**						
Open Water	24.56	13.38	73.71	36.38	20.56	31.70

Satellite classification (% of site) showing pre (2012/13) and post (2014–2017) bund removal.

*Weeds of national significance

†Ponded pasture grasses.

Following bund removal (6^th^ October 2013), vegetation condition of the wetland remained unchanged for the following 3 months. However, the effect of increased EC on the ponded grasses and weeds was noticeable from a few days after the first tidal ingress at the end of January 2014, with a rapid yellowing of the vegetation occurring. Weed death continued after this and by the time of the next remote vegetation survey in August 2014 exotic species reduced to below 20% within the tidally effected area (Table 2). This was driven by large reductions in olive hymenachne (*H*. *amplexicaulis)*, water hyacinth (*E*. *crassipes*) and salvinia (*S*. *molesta)* with only small decreases in Para grass (*U*. *mutica*) and Aleman grass (*E*. *polystachya*) which occurred on the wetland margins. *Nymphaea gigantea* also decreased by two thirds at this point (Fig 17C), (Table 2). A dramatic change is evident (Fig 17C) with most of the exotic vegetation being replaced by open water. The following year (2015) saw 4 tidal periods over 3.7 m with the wetland becoming more saline for an average of 13 days and then brackish for 178 days, again with 3 periods being recorded at the 450 m above bund location. 2015 was the driest year on record for the region, which accounts for the differences in tidal inundation (days), as wetland depth and associated freshwater outflow did not restrict tidal inflow to the same degree as the previous year. Satellite imagery recorded an increase in two of the ponded pastures—Aleman (*E*. *polystachya*) and Para grass (*U*. *mutica*)—invading areas previously inhabited by olive hymenachne (*H*. *amplexicaulis)* this occurred on the wetland margins and comprised ~25% of the site (Fig 17D), (Table 2). Olive hymenachne was reduced to ~5% with salvinia and water hyacinth being undetectable on the remote sensing imagery (Fig 17D), (Table 2). There was an increase in native aquatic plants driven by the appearance of *Eleocharis dulcis* (Bulkuru) within the lower half of the site as well as an increase in *N*. *gigantea* in the upper site. *Spirodela punctata* (Duck weed) was also present (~3.5%) in some of the shallower areas originally inhabited by *H*. *amplexicaulis*. These native species generally took advantage of the disappearance of exotic species and newly available open water. The entire wetland dried out in October 2015 remaining dry for two months (Fig 4A) and then received small flushes of rainfall until March 2016 (pH as low as ~3 occurred for a short period due to acid sulphate soil oxidation and leaching). At this time, as a result of increased wetland accessibility, predation by feral pigs removed most of the native Bulkuru (*E*. *dulcis)*.

**Fig 18:**
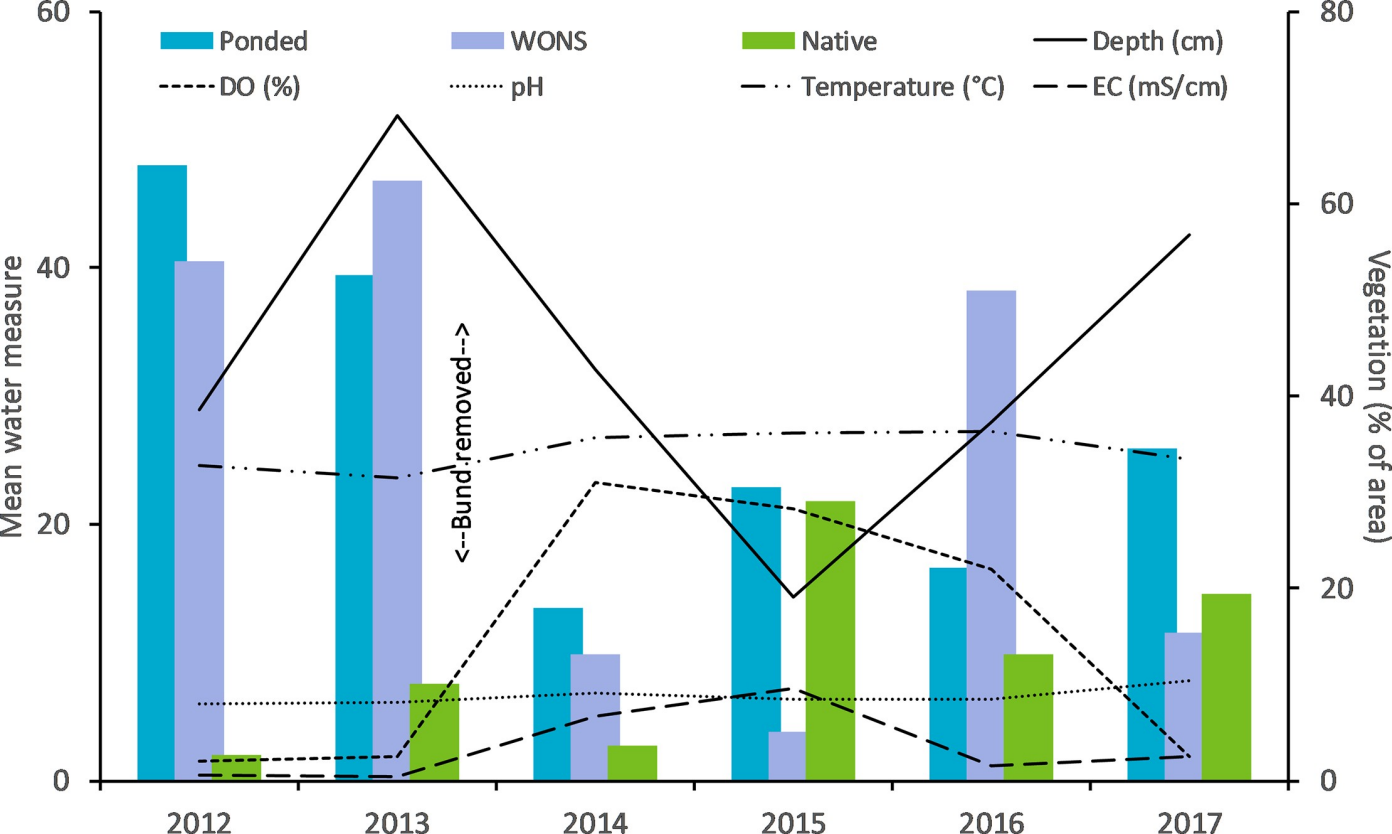
Yearly mean water quality and vegetation proportions. Shown are the mean water quality measures within the above bund location of the Boolgooroo wetland with vegetation grouped into weeds of national significance, Ponded pastures and native vegetation.

There was a large amount of rainfall during March of 2016 with constant rainfall input until July with no tidal influence measured. This period saw the return of *S*. *molesta* (~40% of the area) invading open water and the areas previously inhabited by *E*. *dulcis and N*. *gigantea* in the upper reaches (Fig 17E*)*. *E*. *polystachya* and *U*. *mutica* were reduced slightly from the margins to be replaced by *H*. *amplexicaulis*. Whilst some of the area occupied by *N*. *gigantea* were now used by salvinia, the water lily had become denser (Fig 17E) and remained stable at around ~13% of the Boolgooroo area (Table 2). *E*. *dulcis* was no longer detectable on the imagery after the feral pig predation event of 2016, however some individual plants were observed in the lower Boolgooroo area at this time. Available DO dropped to close to zero after the March 2016 rainfall with increases in salvinia. In the final 7 months of the study (2017) 3 tides affected the wetland, but only to the above 50 m location (Fig 4C) although the spikes in EC from September and December 2016 tides could be considered here in regards to vegetation change as tidal input during this period was observed to drastically reduced the population of salvinia. The 2017 image classification (Fig 17F) clearly shows the reduction in salvinia to marginal locations with *N*. *gigantea* moving into the areas that it previously occupied in the upper Boolgooroo, and the native sedge, Bulkuru, reappearing in the lower Boolgooroo wetland. At this time the saltmarsh species *S*. *litoralis* (Club rush) appeared in a small area (Table 2) directly above the eastern end of the bund location (Fig 17F). Ponded grasses, which had remained on the wetland margins since 2014, showed a further decrease in *H*. *amplexicaulis* extent down to ~5%, this is similar to the 2015 level and most likely representative of the extent of effective weed control of tidal intrusion. Growth of *U*. *mutica* and *E*. *polystachya* increased however inhabiting areas previously occupied by *H*. *amplexicaulis* in 2016. Increase by Aleman grass (*E*. *polystachya*) even more pronounced in this period. Whilst the dataset is limited to only 6 yearly aggregates the relationships between Vegetation and water quality measures (Fig 18) can still be assessed in general terms and with simple linear correlation (Table 3).

**Table 3:** Linear correlation between water quality measures and vegetation proportion.

	WONS	Ponded pasture	Native vegetation
**Depth**	0.31	0.43	-0.37
**Dissolved oxygen**	-0.56	-0.81**	0.22
**pH**	-0.60	-0.46	0.28
**Temperature**	-0.49	-0.85**	0.36
**Electrical conductivity**	-0.86**	-0.47	0.51

**P < 0.05, n = 6

Most notable here are the significant correlations for WONS and Ponded pastures, with WONS decreasing notably with increases in EC (Table 3), (Fig 18 – 2014 and 2015), and increases in DO and Temperature occurring as ponded pastures decline from 2014 to 2016. While not significant statistically the other correlation values in Table 3 do give an indication of the relationship between variables, of note are the positive correlations for native vegetation, DO, pH, Temperature and EC which are in contrast to exotic vegetation results.

### Fish biodiversity

The fish community in Mungalla wetland prior to bund wall removal was of low abundance and diversity in comparison with other coastal freshwater wetlands in the region [52], with only three species recorded (Empire gudgeon, *Hypseleotris compressa*; Eastern rainbow fish, *Melanotaenia splendida inornata*; the invasive Mosquitofish, *Gambusia holbrooki*) despite sampling efforts comprised of a combination of cast nets, bait traps, electrofishing and visual observation [49] (Table 4). The most abundant species was the empire gudgeon (*Hypseleotris compressa*), which is widespread in the region owing to a diadromous movement ecology where it migrates to access upstream and downstream river areas [53]. Presence of these species suggests that they are tolerant of poor water quality (particularly low DO), as has been the case in the wetlands prior to bund wall removal.

**Table 4:** Fish community composition in the Mungalla wetland (Boolgooroo) before bund removal in 2009 and following removal in 2016.

Common name Species	Pre bund removal survey (2009)	Post bund removal survey (2016)
Mosquitofish *Gambusia holbrooki*	**√**	**√**
Hardyhead *Craterocephalus stercusmuscarum*		**√**
Empire gudgeon* Hypseleotris compressa	**√**	**√**
Tarpon *Megalops cyprinoides*		**√**
Rainbowfish *Melanotaenia splendida*	**√**	**√**
Barramundi* Lates calcarifer		**√**
Silverbiddy* Gerres subfasciatus		**√**
Banded scat* Selenotoca multifasciata		**√**
Long finned eel* Anguilla reinhardtii		**√**
**Total**	3	9

*diadromous movement ecology.

Post bund removal fish surveys conducted in May 2016, found the same three species identified before bund removal with an additional six species recorded, although the increased number of species recorded is still low by local standards [52]. The most notable records in the post bund survey were barramundi (*Lates calcarifer*), silver biddy (*Gerres subfasciatus*) and banded scat (*Selenotoca multifasciata*); each of these species have a diadromous movement ecology, requiring access to wetlands to fulfil critical life cycle stages.

## Discussion

The restriction of seawater from coastal wetlands using earth bunds (e.g. dams, dykes or levees) is widespread and has been seen to cause the degradation and/or loss of salt marsh ecosystems worldwide [3, 9, 54]. Prolonged exclusion of seawater leads to the loss of native halophytes and widespread invasions of freshwater species, in addition to major changes in sedimentation rates and soil chemistry [55]. These hydrological changes not only influence larger wetland species, but also microscopic species [56], as well as, more generally, nutrient dynamics and water quality conditions [57, 58]. Recognition that these bunded wetlands are not natural has led to an increasing number of tidal restoration projects [9]. For example, Smith *et al*., [59] describe how freshwater plants are being replaced by native salt marsh plants following the restoration of tidal flows to the Hatches Harbor salt marsh in Cape Cod, Massachusetts, USA. In Australia, tidal flows were reinstated into coastal wetlands in the Hunter estuary in New South Wales and this increased the area of salt marsh, largely through expansion into areas of pasture [28]. Conversely seawater intrusion into coastal wetlands such as the Gippsland lakes in Victoria [60] and the rivers in the Northern Territory [61] has also caused changes in wetland ecology., These cases however, are generally seen as undesirable, since they changed the wetlands from their natural freshwater state. These salinization effects are reported worldwide and are attributed to a range of anthropogenic impacts including sea level rise [62]. In the case of the Boolgooroo wetland on Mungalla station the removal of the earth bund and subsequent reintroduction of tidal water ingress, returning the wetland to its historical halophytic state was a desirable outcome, where the receipt of occasional tidal pulses was enough to assist with naturally supressing invasive aquatic freshwater plant species.

The extent of tidal inundation in coastal floodplains, in terms of duration and frequency, is critical in the delivery of successful restoration outcomes [63]; which has become particularly relevant for managers when considering future climate change [64, 65]. Seawater entry into the Mungalla wetlands does not occur often since, with the bund removed, only the highest of tides, of approximately 3.6 m, are able to penetrate the wetland. These usually occur in sequences of 2 to 3 consecutive days on four occasions during the summer (December to March) and a similar number in winter (June to September). However, if the wetland contains water deeper than ~ 0.4 m a ‘hydraulic barrier’ is formed meaning that tides effectively need to be greater than 3.6 m to penetrate the wetland upstream of the bund wall location. This may not happen frequently as tides in excess of 4.0 m only occur about once a year. Seawater ingress is therefore more likely to happen when the wetland depths are lowest, either during late winter or in years of low summer rainfall (experienced during this research program). However, when hydraulic pressure is low during low rainfall years and in the pre-summer period the frequency and duration of seawater ingress to the Boolgooroo region of the Mungalla wetland can exceed that required to cause permanent damage to the invasive freshwater weeds. Salinity tolerance tests carried out by Reid et al., [66] found that the growth and survival of Aleman grass (*Echinochloa polystachya*), olive hymenachne (*Hymenachne amplexicaulis*), Para-grass (*Urochloa mutica*) and water hyacinth (*Eichhornia crassipes*) were all affected by changes in EC, even when exposed to only 30% seawater concentration for as little as a single day. Although tolerance varied between these four weed species (Aleman grass being most tolerant), each had stopped photosynthesis and mortality rates were very high when exposed to 100% seawater equivalent for longer than 7 days. Clearly removal of the earth bund allowed seawater to effectively penetrate the wetland on multiple occasions creating saline conditions that would be expected to have a marked impact on the freshwater adapted weeds found above the bund location. Indeed, surveys of the wetland vegetation before and after the removal of the bund show that there was a large and relatively rapid reduction in freshwater weeds (primarily olive hymenachne, water hyacinth and salvinia) above the bund location, with the re-emergence of native salt tolerant plants after only 2–3 years. However, since wetland depth and tidal height conditions required to change the wetland vegetation towards a more halophytic composition may only occur in the driest of years (as in 2015), or during dry pre-summer conditions, there is a risk of the wetland reverting to dominance by invasive freshwater species. In fact, this occurred in 2016 with an initial re-invasion of the floating species *S*. *molesta* (salvinia) followed by some encroachment of ponded pastures from the wetland margins. Of great interest, following saltwater ingress, has been the expansion of Aleman grass (*E*. *polystachya*), being the most tolerant to saline conditions [66], which will most likely continue to dominate from the wetland margins inwards, replacing Para grass (*U*. *mutica*) and Hymenachne (*H*. *amplexicaulis*) when conditions are suitable.

Fish accessing coastal transitional waters on floodplains is important for completion of lifecycle stages [13, 67, 68], but this access is generally prohibited in urban, industrial and agricultural areas because of human engineered modification to the natural landscape [69–71]. On floodplains considerable effort has gone into restoring this connectivity for the purposes of reconnecting fish access and extending habitat areas. Here, removing the bund wall resulted in fish biodiversity increasing presumably as a result of amended connectivity between the wetland and the ocean, but also because of improved water quality conditions available for fish when they access the wetland. The very poor water quality in the wetland, especially the extremely low dissolved oxygen which would regularly expose, such as fish (and other aquatic fauna), to acute and chronic hypoxia risks led to low fish numbers in the wetland prior to bund removal. The low oxygen levels before bund removal were likely to have been due to the presence of the dense mat of freshwater weeds that limited oxygen transfer and light penetration to the bottom of the wetland. The challenge of limited oxygen and light availability for plant growth and respiration recovery in waterways is a global problem [72–76]. Butler and Burrows [51] found that there were significant risks of acute exposure when DO was below 30% saturation (the ‘acute trigger value’ ATV). Prior to bund removal hypoxia risks were severe, as DO was below the ATV threshold virtually 100% of the time. With the earth bund removed, DO gradually increased in the first wet season (2013/14), but still fell below the ATV for 93% of the time. The improvement in DO was more dramatic following the 2014 dry season, where DO only fell below the ATV for 49% of the time. Given the normal diurnal cycling of DO at the bottom of a wetland [77], these latter trigger value failures should not present a high risk to fish that can swim towards the surface where DO would be higher [78]. An aquatic ecology survey of the Mungalla wetlands completed 4 years before the bund was removed [78] found that the weed infested sections of the wetland did not provide suitable habitat refugia for most fish species, and created conditions that increase the wetlands’ susceptibility to acute episodic periods of low dissolved oxygen. That study also showed that water quality was better in ‘open’ (weed free) parts of the wetland. This was probably the habitat (22% of the wetland area) where the few species of fish (3 species) existed at that time (May 2009). Reduction of wetland depth following bund removal may also pose a risk to wetland biota via its effect on water temperature. Water temperatures recorded above the Mungalla bund location did not exceed 34°C either before (2013) or after (2014) removal. Therefore, bund removal *per se* did not affect the wetland thermal regime. However, when water depths were very shallow (< 40 cm), as in the 2015 wet season, due to the very low rainfall in that year, temperatures exceeded 31°C over 60% of the time, 34°C for 35% of the time. These higher temperatures could have had a major impact on freshwater aquatic species, if trapped within the wetland, as many tropical fish [79] and freshwater crustaceans [80, 81] cannot survive prolonged exposure to such high temperatures. However, with connectivity improved via bund removal and a reduction of weed species, aquatic biota could move to other parts of the wetland or adjacent creeks during these periods. This is also true for the short periods of low pH when the wetland water became acidic (pH 3–3.5), reaching conditions that could be lethal for aquatic invertebrates and could even kill fish [82, 83]. These conditions follow periods of drought, when the acid sulphate soil in the wetland is exposed to air, releasing a pulse of sulphuric acid [84] when the wetland is initially reflooded by fresh or saline waters. Interestingly, if high tides continue to occur during these periods of low wetland depth, the alkaline seawater ingress can eliminate the acidic conditions; which would not have occurred when the wetland was bunded. However, the buffering of saltwater potential has been shown to contribute to secondary implications, including the precipitation of heavy metals that are available in the water column [8].

Freshwater weed infestation is not only widespread in many of the coastal wetlands in North Queensland [33, 36], but remains a significant problem globally within all freshwater systems [85, 86], with the primary control mechanisms being herbicide and mechanical removal [36, 87, 88]. However, these methods are expensive, can have undesirable ecological consequences, and are only effective for a limited time. In some cases, these methods have been shown to have little to no impact [89]. For example, in the years before bund removal on Boolgooroo chemical spraying with herbicide did increase the open water area, but this mitigation measure provided only a temporary solution with aquatic weeds such as olive hymenachne again present only 2–3 months after spraying and Aleman grass largely unaffected most likely due to herbicide resistance at the application rates used [90]. Although spraying with more ecologically acceptable saline water has been attempted for control of water hyacinth [91] it is not yet widely used for freshwater weed control. With approximately 30% of coastal wetlands being bunded in the Great Barrier Reef region [92], removal of an earth bund or levee could provide a more cost effective and sustainable means of controlling freshwater weeds and improving water quality. However, landholders and government do still need to take care to fully consider tidal boundary laws and amendments when considering ponded pasture reconversion projects [93].

## Conclusions

**Fig 19:**
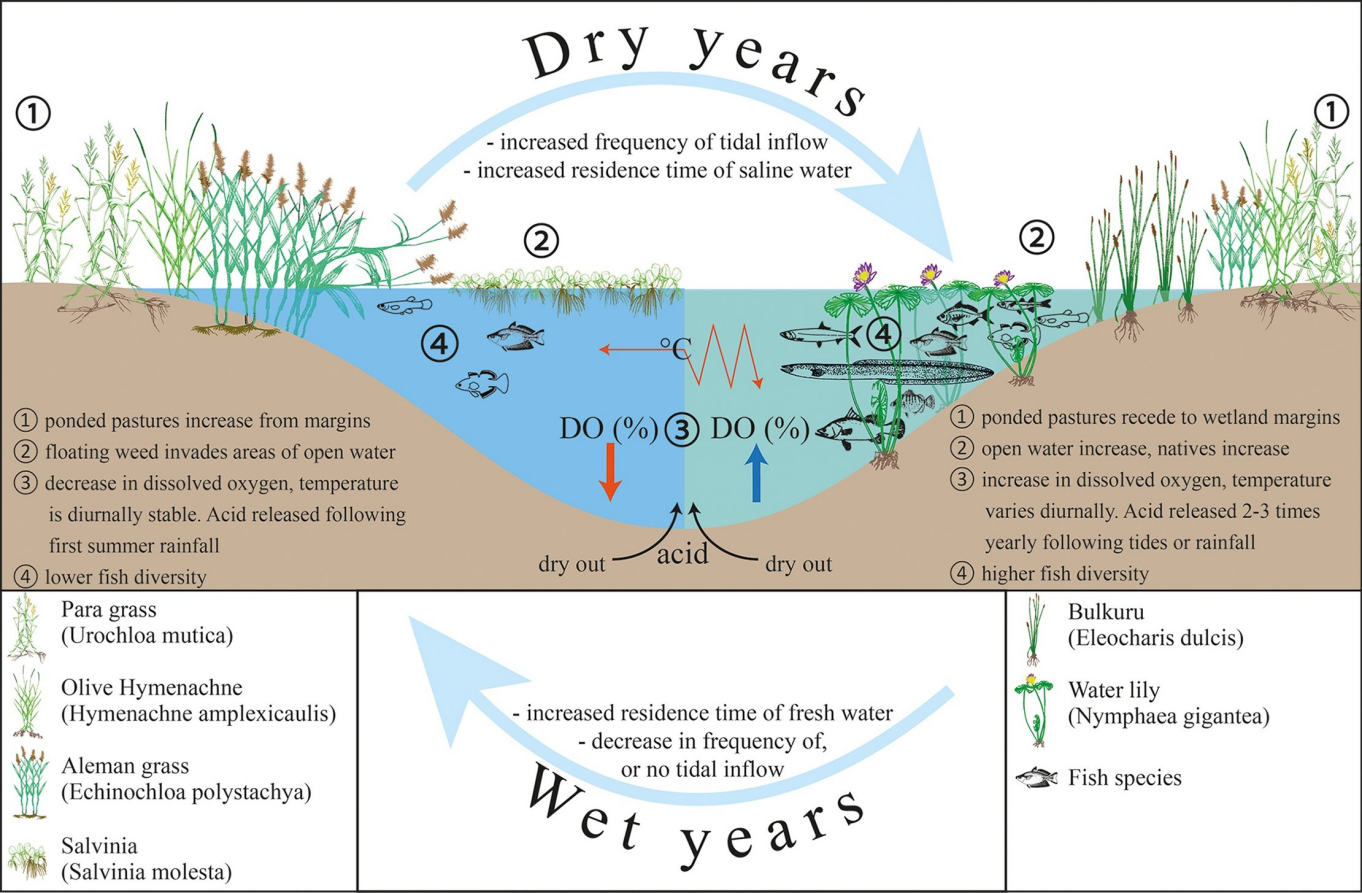
Conceptual model showing changes due to seasonal oscillations within the shallow Boolgooroo region of the Mungalla wetlands. This oscillation/cycle is likely to occur on other shallow wetlands if this method of passive remediation is employed.

With the limited success of control methods to restore wetlands as productive coastal features in the Great Barrier Reef catchment area, this study revealed that reinstatement of tidal flows into bunded estuarine wetlands is relatively effective in the removal of some freshwater weeds and ponded pastures. As a passive remediation method reintroduction of tidal flow is a sustainable, efficient and cost-effective management option for restoring aesthetic and ecological values of coastal wetlands. Surprisingly, gross changes towards a more natural system occurred within a relatively short timeframe, with the reappearance of native vegetation, Water lilies and Bulkuru, improvements in water quality and fish biodiversity taking less than 3 years. However, the weight of evidence presented here after 5 years of monitoring, shows that the abundance of native and invasive plant species appears to oscillate depending on seasonal rainfall which can induce hydrologic pressure to repress tidal water ingress, which in turn drives dissolved oxygen and temperature regimes (through vegetation and depth changes) within the wetland, affecting fish occupancy. These changes are modelled conceptually in Fig 19, describing what is happening on the Mungalla wetland post-bund removal, showing the oscillation or cycle between freshwater and saltwater tolerant plant species, associated water quality, and fish presence, associated with the preceding years’ weather conditions (primarily summer rainfall).

Variation in wetland salinity, due to tidal intrusion, and related species and water quality variation described here may be viewed as a short term effect with the wetland reaching an equilibrium similar to other local natural estuarine wetlands in the longer term, i.e. Bulkuru dominant toward the seaward end of the wetlands along with other saltmarsh-adapted species. Vegetation in the upper reaches will most likely remain primarily freshwater adapted species, with tidal influence in only very dry years (such as 2015). A particularly interesting outcome from this research has been the replacement of other ponded pastures by Aleman grass (*Echinochloa polystachya*). Aleman grass appears to be tolerant of marginally brackish conditions–giving it the ability to reinvade on years of lower tidal ingress, and has already shown some herbicide resistance [90]. Further research is necessary to understand more around the effects of saltwater impact on Aleman grass, including a combination of herbicide and seawater treatment, as removal of this grass may become the next challenge.

Whilst reinstatement of tidal flow has been successfully applied elsewhere to restore ecological function, this study appears to be the first of its kind targeting wetland weeds and specifically ponded pastures in the Great Barrier Reef region, and as such is an important case study for similar restoration efforts needed to effect reef water quality and the Australian government’s plan of coastal wetland restoration and protection under the Reef 2050 plan[94].
